# Tripartite motif 8 promotes the progression of hepatocellular carcinoma via mediating ubiquitination of HNF1α

**DOI:** 10.1038/s41419-024-06819-y

**Published:** 2024-06-15

**Authors:** Yu Peng, Hui Qian, Wen-Ping Xu, Meng-Chao Xiao, Chen-Hong Ding, Fang Liu, Huan-Yu Hong, Shu-Qing Liu, Xin Zhang, Wei-Fen Xie

**Affiliations:** 1 Department of Gastroenterology, Changzheng Hospital, Naval Medical University, Shanghai, China; 2 Department of Gastroenterology, Shanghai East Hospital, School of Medicine, Tongji University, Shanghai, China

**Keywords:** Oncogenes, Transcription, Biomarkers

## Abstract

Tripartite motif 8 (TRIM8) is an E3 ligase that plays dual roles in various tumor types. The biological effects and underlying mechanism of TRIM8 in hepatocellular carcinoma (HCC) remain unknown. Hepatocyte nuclear factor 1α (HNF1α) is a key transcriptional factor that plays a significant role in regulating hepatocyte differentiation and liver function. The reduced expression of HNF1α is a critical event in the development of HCC, but the underlying mechanism for its degradation remains elusive. In this study, we discovered that the expression of TRIM8 was upregulated in HCC tissues, and was positively correlated with aggressive tumor behavior of HCC and shorter survival of HCC patients. Overexpression of TRIM8 promoted the proliferation, colony formation, invasion, and migration of HCC cells, while TRIM8 knockdown or knockout exerted the opposite effects. RNA sequencing revealed that TRIM8 knockout suppresses several cancer-related pathways, including Wnt/β-catenin and TGF-β signaling in HepG2 cells. TRIM8 directly interacts with HNF1α, promoting its degradation by catalyzing polyubiquitination on lysine 197 in HCC cells. Moreover, the cancer-promoting effects of TRIM8 in HCC were abolished by the HNF1α-K197R mutant in vitro and in vivo. These data demonstrated that TRIM8 plays an oncogenic role in HCC progression through mediating the ubiquitination of HNF1α and promoting its protein degradation, and suggests targeting TRIM8-HNF1α may provide a promising therapeutic strategy of HCC.

## Introduction

Liver cancer is the sixth most common malignant tumor and the third leading cause of cancer-related death worldwide in 2020 [1]. Hepatocellular carcinoma (HCC), accounting for 80% ~90% of the cases, is the most common type of liver cancer and represents a major global public health challenge [2]. Although the treatment options of HCC are more diversified than ever and the prognosis for HCC patients has been improved, the early metastasis and frequent recurrence lead to a poor 5-year survival rate of advanced HCC [3, 4]. The emergence of precision medicine and targeted therapy shed new light on the treatment of HCC. Hence, investigating the underlying molecular mechanism of HCC and identifying novel therapeutic targets are urgently needed to improve the survival of patients with HCC.

Protein ubiquitination is a vital and dynamic post-translational modification that regulates a series of biological functions including autophagy, proteasome degradation and signal transduction [5, 6]. Substantial evidence has demonstrated that the ubiquitin-proteasome system (UPS) is often dysregulated in tumorigenesis, leading to abnormal protein accumulation or enhanced protein degradation [7]. E3 ligases play a crucial role in transferring ubiquitin from E2 conjugating enzymes to specific substrates, thereby conferring ubiquitination specificity [8]. Elevated levels of E3 ligases have been widely identified to cause abnormal UPS-mediated protein degradation in tumorigenesis [9]. The tripartite motif-containing (TRIM) family proteins are characterized by a RING-finger domain, one or two B-boxes motifs, and a coiled-coil domain [10]. Notably, the activity of E3 ligases of most TRIM members is conferred by the RING finger domain. Tripartite motif 8 (TRIM8), a member of TRIM family possessing E3 ligase activity, has been implicated in the regulation of substrate stability and function by mediating K63-linked or K48-linked polyubiquitination [11]. A dual role of TRIM8 in cancers as an oncogene or a tumor suppressor gene has been elaborated in previous studies [11–13]. TRIM8 acts as an oncogene in glioblastoma and Ewing sarcoma, but suppresses tumor growth in breast cancer and colorectal cancer [11, 14]. However, the roles and underlying mechanisms of TRIM8 in HCC have not been reported.

Hepatocyte nuclear factor 1α (HNF1α) is a key transcription factor that is mainly expressed in the liver, and plays a critical part in hepatocyte differentiation, metabolic regulation, and maintenance of liver function [15, 16]. Our previous studies demonstrated that HNF1α expression is significantly reduced in fibrotic liver than in normal liver [17]. We also found that the expression of HNF1α is lower in 70% of HCC tissues than that in paracancerous tissues [18]. Consistently, it was reported that the level of HNF1α protein is reduced in poorly differentiated HCC samples compared with well differentiated HCC tissues [19]. These findings revealed that reduced expression of HNF1α is a critical event in the development and progression of HCC. More importantly, it has also been demonstrated that up-regulation of HNF1α significantly suppressed the malignant phenotypes of HCC cells and induced the transformation of HCC cells into normal hepatocytes [18, 20], suggesting reverse the downtrend of HNF1α is a potential strategy for HCC therapy. Nonetheless, to date, the upstream mechanism of the reduction of HNF1α has been largely unknown.

In this study, we found that TRIM8 interacted with and enhanced the ubiquitination of HNF1α, thus accelerating the degradation of HNF1α and promoting HCC progression. We also demonstrated that TRIM8 functions as an oncogene and might serve as a promising diagnostic and therapeutic target for HCC. Approaches designed to targeting the TRIM8-mediated degradation of HNF1α might improve survival of patients with HCC.

## Results

### Upregulation of TRIM8 is associated with more aggressive characteristics and poorer prognosis of human HCC

**Fig. 1:**
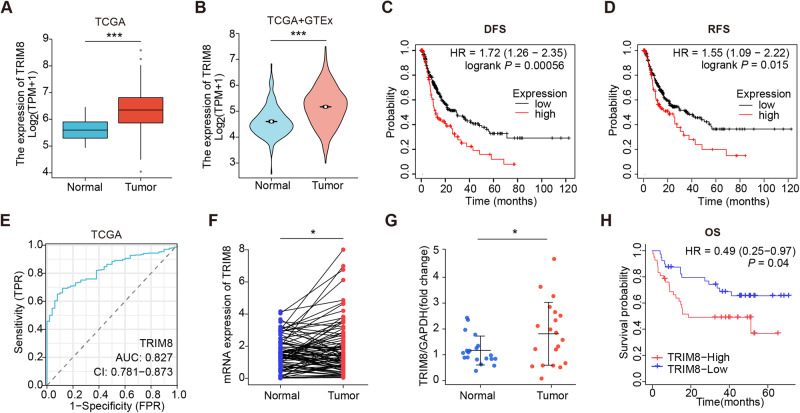
The higher expression of TRIM8 in HCC is related to poorer prognosis of HCC patients. **A** The expression of TRIM8 in HCC tissues (T; *n* = 424) and adjacent non-tumor tissues (N; *n* = 50) in the TCGA database. ***P < 0.001. Welch t’ test. **B** Statistical analysis of TRIM8 expression in HCC tissues (T; *n* = 371) and adjacent non-tumor tissues (N; *n* = 50) in the TCGA database, and in normal liver tissues (N; *n* = 110) in GTEx database. ***P < 0.001. Welch t’ test. **C**, **D** Kaplan–Meier curve and the log-rank test were used to determine the diseases-free survival (DFS) and recurrence-free survival (RFS) in HCC patients with high or low TRIM8 expression based on TCGA database (http://www.kmplot.com/analysis/). **E** The ROC curve of TCGA-LIHC according to the expression of TRIM8. **F** RT-qPCR analyses of TRIM8 mRNA in paired tumor tissues (tumor) and adjacent non-tumor liver tissues (normal) from 85 HCC patients. *P < 0.05, Wilcoxon matched pairs test. **G** Semi-quantification of TRIM8 protein expression detected by Western blotting analysis in paired tumor tissues (tumor) and adjacent non-tumor liver tissues (normal) from 20 HCC patients. *P < 0.05, Wilcoxon matched pairs test. **H** Overall survival (OS) compared between 82 patients with HCC presenting low (*n* = 41) or high TRIM8 (*n* = 41) mRNA expression based on the cut-off value of the median value of TRIM8 using the log- rank test.

We firstly searched the datasets from the Cancer Genome Atlas (TCGA) for the TRIM8 transcript levels in HCC. The results showed that the expression of TRIM8 was significantly upregulated in the HCC tissues compared with the surrounding non-tumor liver tissues and normal liver tissues (Fig. 1A, B). Searching in www.kmplot.com for the prognostic value of TRIM8 in patients with HCC, we found that higher TRIM8 level correlated with a shorter disease-free survival (DFS) and recurrence-free survival (RFS) (Fig. 1C, D). Receiver operating characteristic curve (ROC) was used to verify the diagnostic ability of TRIM8 in TCGA-LIHC cohort and resulted in an area under the curve (AUC) of 0.827, indicating TRIM8 can serve as a candidate diagnostic biomarker for HCC (Fig. 1E). To further validate the clinical significance of TRIM8 expression in HCC, we detected the levels of TRIM8 in 85 pairs of HCC and paracancerous tissues using RT-qPCR. TRIM8 mRNA levels were also elevated in the HCC tissues compared with the paracancerous tissues in this cohort (Fig. 1F). Consistently, the protein expression level of TRIM8 was markedly upregulated in HCC tissues (Fig. 1G; Supplementary Fig. S1A–C). Interestingly, clinicopathological analysis clarified that upregulation of TRIM8 in human HCC was significantly correlated with aggressive clinical and pathological characteristics including less tumor capsule (*P* = 0.0176), more portal vein tumor thrombus (PVTT) (*P* = 0.0156), more advanced Barcelona Clinic Liver Cancer (BCLC) stages (*P* = 0.0404) (Table 1). Correspondingly, higher TRIM8 levels in these HCC samples also correlated with a shorter overall survival (OS) of HCC patients (Fig. 1H). These data demonstrate that TRIM8 expression is clinically correlated with outcomes of HCC patients.

**Table 1:** Correlation between the TRIM8 expression and the clinicopathologic characteristics of HCC patients.

Characteristics	TRIM8-High (*n* = 41)	TRIM8-Low (*n* = 41)	*P* value^a^
Age, *n* (%)			0.5936
≤60	31 (37.8%)	33 (40.2%)	
>60	10 (12.2%)	8 (9.8%)	
Gender, *n* (%)			0.4997
Male	35 (42.7%)	37 (45.1%)	
Female	6 (7.3%)	4 (4.9%)	
HBV infection, *n* (%)			0.1139
Yes	35 (42.7%)	40 (48.8%)	
No	6 (7.3%)	1 (1.2%)	
Yes	23 (29.9%)	18 (23.4%)	
AFP (ug/L), *n* (%)			0.2689
≤400	22 (26.8%)	17 (20.7%)	
>400	19 (23.2%)	24 (29.3%)	
Tumor size(cm), *n* (%)			0.7853
>5	33 (40.2%)	32 (39%)	
≤5	8 (9.8%)	9 (11%)	
Tumor capsule, *n* (%)			0.0176
Yes	23 (28%)	33 (40.2%)	
No	18 (22%)	8 (9.8%)	
MVI, *n* (%)			0.6539
No	16 (19.5%)	18 (22%)	
Yes	25 (30.5%)	23 (28%)	
PVTT, *n* (%)			0.0156
No	21 (25.9%)	32 (39.5%)	
Yes	19 (23.5%)	9 (11.1%)	
Recurrence, *n* (%)			0.3500
No	10 (18.2%)	18 (32.7%)	
Yes	13 (23.6%)	14 (25.5%)	
Metastasis, *n* (%)			0.3331
No	23 (35.4%)	23 (35.4%)	
Yes	7 (10.8%)	12 (18.5%)	
TNM Stage, *n* (%)			0.8244
I + II	18 (22%)	19 (23.2%)	
III + IV	23 (28%)	22 (26.8%)	
BCLC Stage, *n* (%)			0.0404
A + B	21 (25.6%)	30 (36.6%)	
C	20 (24.4%)	11 (13.4%)	

The median value of all 82 HCC samples was chosen as the cut-off point separating HCC patients with high TRIM8 expression and low TRIM8 expression.

*MVI* microvascular invasion, *PVTT* portal vein tumor thrombus, *BCLC* Barcelona Clinic Liver Cancer staging.

^a^Chi-square test.

### TRIM8 enhances the malignant properties of HCC cells

**Fig. 2:**
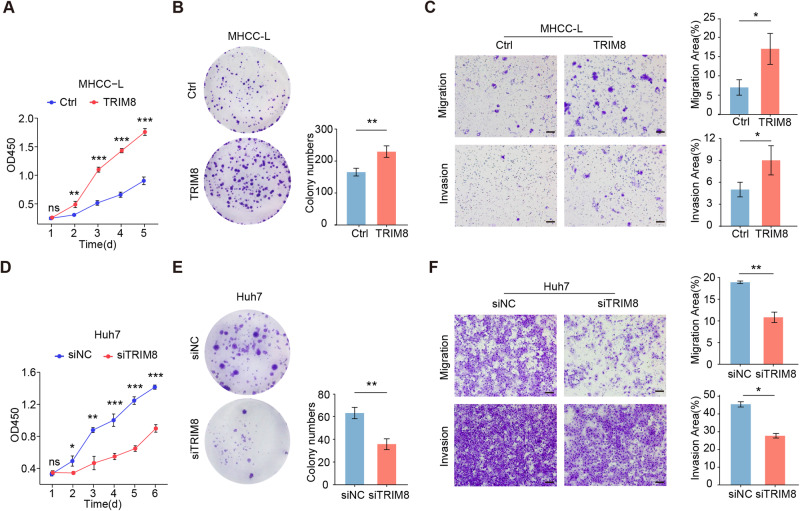
TRIM8 promotes the malignant properties of hepatocellular carcinoma cells in vitro. **A** The proliferation of MHCC-L cells infected with lentivirus expressing TRIM8 (TRIM8) or the control virus (Ctrl) were detected by CCK8 assays. **B** Representative images (left) of colony formation assays and the statistical results (right) of MHCC-L cells infected with lentivirus expressing TRIM8 or the control virus. **C** Migration (top) and invasion (bottom) assays of MHCC-L cells infected with lentivirus expressing TRIM8 or the control virus. **D** The small interfering RNA targeting TRIM8 (siTRIM8) inhibited the proliferation of Huh7 cells. **E** siTRIM8 suppressed the colony formation ability of Huh7 cells. **F** Transwell assays showed the reduced migration and invasion of Huh7 cells transfected with siTRIM8, as compared with Huh7 cells transfected with the negative control (siNC). Scale bars = 50 μm. Experiments were performed in triplicate and data are presented as means ± SEM. *P < 0.05, **P < 0.01 and ***P < 0.001 using two- tailed Student’s *t* tests.

To identify the undetermined effects of TRIM8 on the malignant properties of human HCC cell lines, we firstly detected the mRNA and protein expression of TRIM8 in six HCC lines and a normal hepatocyte line L02. The results showed that TRIM8 levels were significantly increased in all of the detected six HCC cell lines relative to L02 (Supplementary Fig. S2A, B). Then, we reinforced the expression of TRIM8 in MHCC-L cells with the lowest endogenous TRIM8 protein level using lentivirus, and reduced the expression of TRIM8 in Huh7 cells with the highest endogenous TRIM8 protein level using small interfering RNA (siRNA) targeting TRIM8 (Supplementary Fig. S2C–F). Overexpression of TRIM8 significantly promoted the proliferation, colony formation, migration and invasion of HCC cells in vitro (Fig. 2A–C). In contrast, inhibition of TRIM8 decreased the malignant phenotypes of HCC cells (Fig. 2D–F). To further explore the oncogenic role of TRIM8 in hepatoma cells, TRIM8 gene knockout (TRIM8-KO) HepG2 cells were constructed using CRISPR-Cas9 technology (Supplementary Fig. S2G). Consistent with siRNA-mediated effects, knockout of TRIM8 significantly suppressed the proliferation and colony-formation of HepG2 cells (Supplementary Fig. S2H, I). Based on these data, TRIM8 functions as a mighty oncogene in HCC.

### TRIM8 regulates cancer-related pathways in HCC

**Fig. 3:**
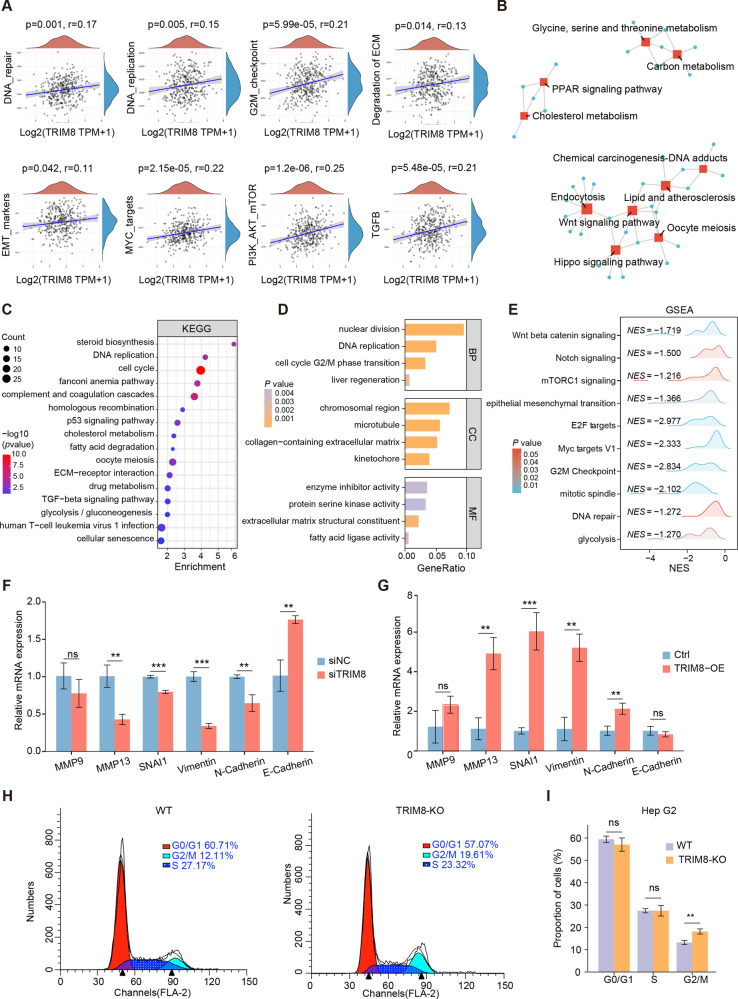
Determination of TRIM8-associated signaling pathways in HCC. **A** Spearman correlation analysis between TRIM8 and pathway scores according to the ssGSEA algorithm. The signal pathway positively correlated with the high expression of TRIM8 in HCC samples was screened. **B** Gene correlation analysis screened out 100 TRIM8-related genes based on TCGA-LIHC dataset for KEGG analysis, and shown as network diagram. **C**, **D** KEGG and GO pathway enrichment analyses of DEGs from WT and TRIM-KO HepG2 cells. *n* = 3 samples per group. **E** GSEA analysis of RNA-seq data in WT and TRIM-KO HepG2 cells. The mRNA expression of EMT-associated factors in Huh7 cells transfected with siTRIM8 or siNC (**F**) and in MHCC-L cells infected with lentivirus expressing TRIM8 (TRIM8-OE) or the control virus (Ctrl) (**G**) are shown. **H** Flow cytometry analyses were performed to evaluate the impact of TRIM8 knockout on the distribution of cell cycle phases. **I** Statistical results of flow cytometry analyses. **F**–**I** Experiments were performed in triplicate and data are presented as means ± SEM. ns no significance, **P < 0.01 and ***P < 0.001 using two- tailed Student’s *t* tests.

To explore the underlying mechanism of TRIM8 in HCC, we performed single sample Gene Set Enrichment Analysis (ssGSEA) to detect which pathways were enriched in TRIM8 high expression samples by utilizing TCGA database. As shown in Fig. 3A, various cancer-related pathways including DNA repair, DNA replication, G2/M checkpoint, degradation of ECM, EMT markers, MYC targets, PI3K-AKT-mTOR and TGF-β pathways were enriched. Then, we performed a gene correlation analysis to select the top 50 genes positive and negative related to TRIM8 respectively, and conducted Kyoto Encyclopedia of Genes and Genomes (KEGG) analysis which revealed that TRIM8-related genes were in involved in Wnt, Hippo, and PPAR signaling pathway as well as in lipid and cholesterol metabolism-related pathways (Fig. 3B). Furthermore, RNA sequencing (RNA-Seq) was conducted to obtain the differentially expressed genes (DEGs) between TRIM8-KO and WT HepG2 cells (Supplementary Fig. S3A, B). KEGG analysis of the DEGs showed a notable enrichment of the cell cycle, cellular senescence, DNA replication, steroid biosynthesis and drug metabolism pathway (Fig. 3C). Gene Ontology (GO) analysis certified that these DEGs were closely related to the process of nuclear division, DNA replication, cell cycle G2/M transition, and extracellular matrix (Fig. 3D). Consistently, Gene Set Enrichment Analysis (GSEA) also indicated that Wnt/β-catenin, Notch, and mTORC1signaling, epithelial mesenchymal transition (EMT) pathway, G2/M checkpoint, DNA repair and glycolysis pathway were inhibited when TRIM8 was knocked out (Fig. 3E).

Further results indicated that EMT-associated factors, including matrix metalloprotein 9 (MMP9), matrix metalloprotein 10 (MMP10), Snai1, Vimentin and N-cadherin were strengthened in the TRIM8-OE groups but inhibited when TRIM8 was knocked down (Fig. 3F, G). It has been reported that silencing of TRIM8 results in a delay of the mitosis progression with a cell accumulation in G2//M phase in HeLa cells [21]. In line with this study, flow cytometry analysis performed in our study also revealed that a higher proportion of HepG2 cells in the TRIM8-KO group were arrested in the G2/M phase (Fig. 3H, I). In conclusion, these results indicated that TRIM8 functions as an important regulator of EMT and cell cycle in HCC progression.

### TRIM8 interacts with HNF1α and negatively regulates its function in HCC cells

**Fig. 4:**
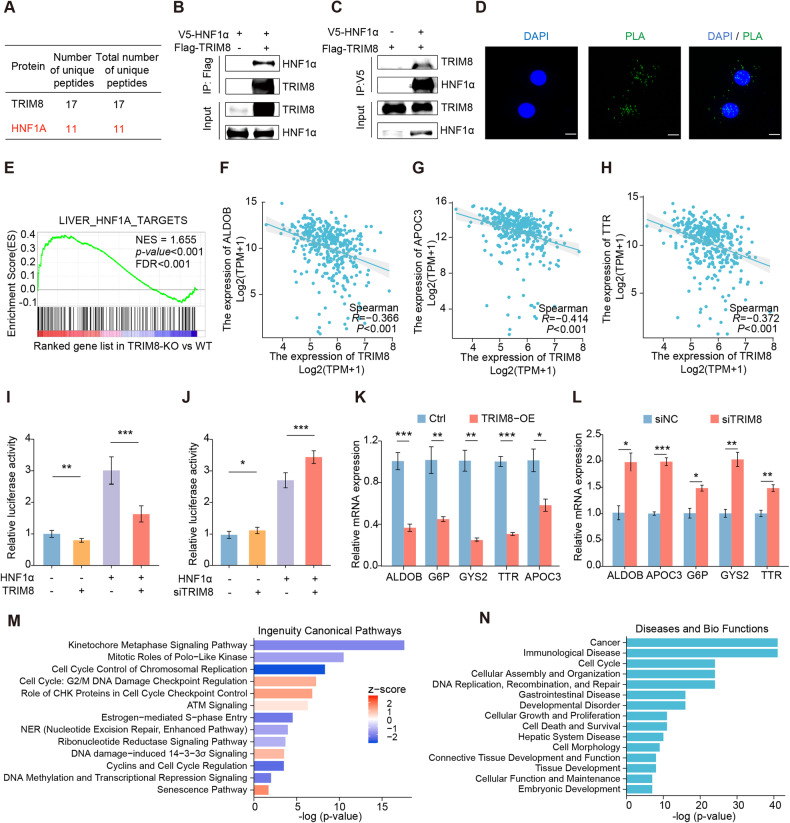
TRIM8 interacts with HNF1α and inhibits the transcriptional activity of HNF1α. **A** Mass spectrometry analysis revealed the potential interaction between HNF1α and TRIM8. **B**, **C** Co-IP assays were performed to detect the interaction between exogenous HNF1α and TRIM8 in HEK293T cells transfected with the plasmids expressing V5-HNF1α and Flag-TRIM8. **D** In situ proximity ligation assay was performed to detect the interaction between HNF1α and TRIM8 in Huh7 cells. Scale bars = 50 μm. **E** GSEA analysis of our acquired RNA sequencing data showed an enrichment of HNF1α target genes in the TRIM8-KO groups. Gene correlation analysis between TRIM8 and HNF1α target genes including ALDOB (**F**), APOC3 (**G**) and TTR (**H**) based on TCGA-LIHC database. Luciferase reporter assays were performed to evaluate the activity of the HNF1α promoter in Huh7 cells transfected with the plasmid overexpressing TRIM8 (**I**) or siTRIM8 (**J**). Relative mRNA levels of HNF1α target genes in MHCC-L cells infected with control lentivirus or lenti-TRIM8 (**K**) and MHCC-L cells transfected with control siRNA or siTRIM8 (**L**). **M** IPA analysis showed the ingenuity canonical pathways of DEGs which were upregulated both in HNF1α-overexpressing cells and in TRIM8-knockout cells. **N** IPA analysis showed the diseases and bio functions of DEGs which were upregulated both in HNF1α-overexpressing cells and in TRIM8-knockout cells. **I**–**L** Experiments were performed in triplicate and data are presented as means ± SEM. *P < 0.05, **P < 0.01 and ***P < 0.001 using two- tailed Student’s *t* tests.

To elucidate the molecular mechanisms of TRIM8 in HCC, we conducted Co-immunoprecipitation (Co-IP) and mass spectrometry (MS) to identify potential substrates of TRIM8. MS analysis revealed that HNF1α, which has been reported to inhibit HCC progression [18, 19], was a potential interaction protein of TRIM8 (Fig. 4A; Supplementary Table 3). We then conducted co-immunoprecipitation (Co-IP) assays to determine whether TRIM8 interacted with HNF1α. The interaction of exogenous TRIM8 and HNF1α was validated in 293T cells transfected with the plasmids expressing Flag-TRIM8 and V5-HNF1α (Fig. 4B, C), while the interaction of endogenous TRIM8 and HNF1α was confirmed in Huh7 cells (Supplementary Fig. S3C, D). The proximity ligation assay (PLA) further identified TRIM8 and HNF1α were located in close proximity to each other in Huh7 cells (Fig. 4D).

To investigate the functional significance of the interaction between TRIM8 and HNF1α, we analyzed the expression of HNF1α target genes by utilizing RNA sequencing data. Interestingly, GSEA analysis revealed that the reported HNF1α target genes [22] were markedly enriched at TRIM8-KO groups (Fig. 4E). Similarly, ninety-four genes of the reported HNF1α target genes were included in the differentially expressed genes from TRIM8-KO RNA-seq analysis (Supplementary Fig. S3E). Furthermore, data from TCGA indicated that the expression of TRIM8 was negatively correlated with the levels of HNF1α target genes, including aldolase B (ALDOB), apolipoprotein C3 (APOC3) and transthyretin (TTR) in human HCC (Fig. 4F–H). Luciferase reporter assays showed that TRIM8 significantly inhibited the transcriptional activity of endogenous and exogenous HNF1α in Huh7 cells (Fig. 4I, J). RT-qPCR analyses confirmed that overexpression of TRIM8 inhibited the expression of HNF1α target genes in HCC cells, while reduction of TRIM8 exerted the opposite functions (Fig. 4K, L). We have previously conducted a microarray analysis to obtain expression profiles in which HNF1α was overexpressed [23]. The Ingenuity Pathway Analysis (IPA) was performed using the DEGs which were upregulated both in HNF1α-overexpressing cells and in TRIM8-knockout cells. In regard to the pathways, these DEGs were mainly associated with the cell cycle, checkpoint regulation, DNA damage and repair, senescence pathways (Fig. 4M), which play important roles in the pathogenesis of various cancers. Moreover, the DEGs are involved in the regulation of diseases and functions including cancer, hepatic system disease, cell death and survival (Fig. 4N). Taken together, these findings indicate that TRIM8 interacts with HNF1α and this interaction may play crucial parts in the progression of HCC.

### TRIM8 facilitates the degradation of HNF1α by the ubiquitin-proteasome system

**Fig. 5:**
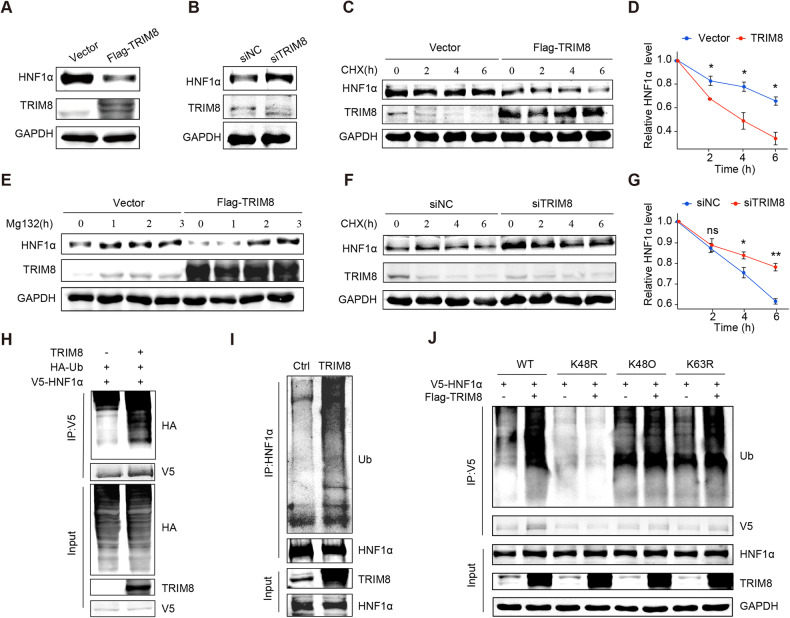
TRIM8 promotes the K48-linked ubiquitination and proteasomal degradation of HNF1α. The expressions of HNF1α protein were detected in the Huh7 cells transfected with the plasmid overexpressing TRIM8 (Flag-TRIM8) and the control vector (**A**) or the Huh7 cells transfected with siTRIM8 and siNC (**B**). **C** Huh7 cells were infected with control or Flag-TRIM8 plasmids and then treated with cycloheximide (CHX; 20 μg/mL) for 0 h, 2 h, 4 h, and 6 h. WB analysis was performed to evaluate HNF1α protein levels. **D** Semi-quantification analysis of HNF1α protein levels after TRIM8 overexpression based on CHX-treated assay. **E** WB analysis of HNF1α in Huh7 cells after treatment with MG132 (20 µM) for 0 h, 1 h, 2 h, and 3 h. **F** Huh7 cells were infected with siNC or siTRIM8 and then treated with CHX (20 μg/mL) for 0 h, 2 h, 4 h, and 6 h, and WB was performed to evaluate HNF1α protein levels. **G** Semi-quantification analysis of HNF1α protein levels after TRIM8-knockdown based on CHX-treated assay. **H** MG132 (20 µM) was applied to Huh7 cells transfected with V5-HNF1α, HA-Ub, TRIM8 plasmids or control plasmids, and 8 h later, ubiquitination assays were performed to determine the ubiquitination levels of HNF1α. **I** Ubiquitination assays determined the ubiquitination of endogenous HNF1α in Huh7 cells transfected with plasmid overexpressing TRIM8 or the control plasmid. **J** The ubiquitination levels of HNF1α after TRIM8 overexpression was examined in Huh7 cells co-transfected with wild-type (WT) and mutated ubiquitin (K48O, K48R, K63R) plasmids. Experiments were performed in triplicate and data are presented as means ± SEM. ns no significance, *P < 0.05 and **P < 0.01 using two- tailed Student’s *t* tests.

To explore the resultant events of the interaction of TRIM8 and HNF1α, we detected the mRNA and protein expression of HNF1α in Huh7 cells transfected with TRIM8 overexpressing plasmid or si-TRIM8, respectively. The results indicated that HNF1α protein level was reduced in the TRIM8 overexpressing group and increased in the si-TRIM8 group, while the HNF1α mRNA levels stayed unchanged in the both groups (Fig. 5A, B; Supplementary Fig. S4A, B). Likewise, the protein levels of HNF1α exhibited a strong increase in TRIM8-KO cells (Supplementary Fig. S4C). Moreover, a lower HNF1α expression was observed in HCC tissues with higher TRIM8 expression (Fig. S4D). These results suggest that TRIM8 reduced the protein of HNF1α. Considering the characteristic of TRIM8 as an E3 ligase, we supposed that TRIM8 promotes the degradation of HNF1α. As shown in Fig. 5C, TRIM8 significantly shortened the half-life of HNF1α in Huh7 cells when treated with cycloheximide (CHX), a protein synthesis inhibitor. The degradation rate of HNF1α in TRIM8 overexpression group was significantly accelerated (Fig. 5D). Furthermore, we observed a significant rebound in HNF1α protein levels upon treatment with Mg132, a proteasome inhibitor, in TRIM8 overexpressing Huh7 cells (Fig. 5E). Notably, Mg132 but not chloroquine, a lysosome inhibitor, blocked the decrease of HNF1α induced by TRIM8 overexpression (Supplementary Fig. S4E). In addition, the degradation of HNF1α in Huh7 cells significantly slowed down when TRIM8 was downregulated (Fig. 5F, G). Subsequently, we observed that the ubiquitination of exogenous and endogenous HNF1α was enhanced by TRIM8 (Fig. 5H, I). Moreover, we demonstrated that TRIM8 promotes the K48-linked ubiquitination of HNF1α (Fig. 5J). To clarify the molecular basis of the interaction between TRIM8 and HNF1α, deletion mutants of TRIM8 were established and Co-IP assay revealed that the RFP-like domain, the C-terminal domain of TRIM8, was involved in the interaction of TRIM8 and HNF1α, but the deletions of the RING, B-box, and coiled-coil domains hardly affected its binding to HNF1α (Supplementary Fig. S5A, B). Furthermore, we determined that deletion of RFP-like domain in TRIM8 abolished HNF1α ubiquitination and degradation (Supplementary Fig. S5C). Collectively, these data suggested that TRIM8 mediates the degradation of HNF1α through the ubiquitin-proteasome pathway.

### TRIM8 mediates the ubiquitination of HNF1α at Lys197

**Fig. 6:**
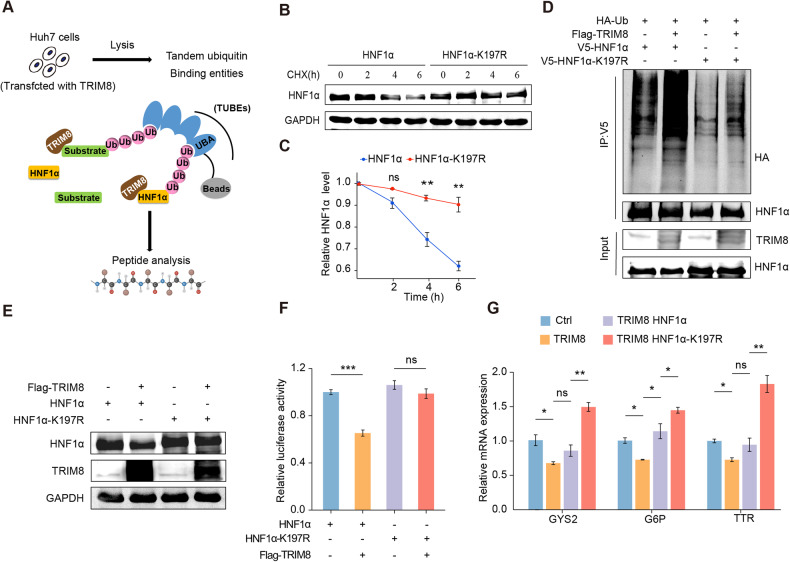
TRIM8 promotes the ubiquitination of HNF1α at Lys197. **A** Schematic illustration of tandem ubiquitin-binding entity (TUBE) pull-down assay. **B** Huh7 cells transfected with V5-HNF1α or V5-HNF1α-K197R plasmids were treated with CHX (20 µg/ml) for 0, 2, 4, and 6 h, and the expression of HNF1α was analyzed by Western blotting (WB) assays. **C** Semi-quantification analysis of HNF1α protein levels in the indicated groups. **D** Ubiquitination assays were performed to determine the ubiquitination levels of HNF1α in Huh7 cells transfected with V5-HNF1α or V5-HNF1α-K197R and HA-Ub plasmids together with Flag-TRIM8 plasmids or negative control plasmids. **E** WB analysis of HNF1α protein levels in Huh7 cells transfected with HNF1α or HNF1α-K197R together with TRIM8 or its control plasmids. **F** Luciferase reporter assays were performed to evaluate the activity of the HNF1α promoter in Huh7 cells transfected with the indicated plasmids. **G** Relative mRNA levels of HNF1α target genes in MHCC-L cells infected with control lentivirus or lenti-TRIM8 together with lentivirus expressing HNF1α or HNF1α-K197R. Experiments were performed in triplicate and data are presented as means ± SEM. ns no significance, *P < 0.05, **P < 0.01, ***P < 0.001 using two- tailed Student’s *t* tests.

To identify the TRIM8-mediated ubiquitination site of HNF1α, we overexpressed TRIM8 in Huh7 cells and isolated ubiquitylated proteins using the Tandem Ubiquitin Binding Entity (TUBE) kit, which has a high affinity for ubiquitin (Fig. 6A; Supplementary Table 4). Subsequently, we used MS analysis to identify these proteins and found that HNF1α was markedly ubiquitinated at lysine residue 197 (Supplementary Fig. S6A, B). Thus, we constructed the plasmid containing an HNF1α lysine-to-arginine mutation at position 197 (K197R). Interestingly, K197R mutation delayed HNF1α degradation in CHX-treated Huh7 cells (Fig. 6B, C). Further ubiquitination assays revealed that K197R mutation significantly reduced the ubiquitination of HNF1α and inhibited TRIM8-mediated ubiquitination of HNF1α (Fig. 6D). In addition, K197R mutation prevented HNF1α degradation in TRIM8-overexpressed Huh7 cells (Fig. 6E). Moreover, luciferase assays revealed that K197R significantly restored the TRIM8-mediated inhibition of HNF1α transcriptional activity (Fig. 6F). Consistently, the inhibition of the HNF1α target genes expression by TRIM8 was reversed by HNF1α-K197R mutant (Fig. 6G). Overall, TRIM8 mediates the ubiquitination of HNF1α at Lys197.

### Blocking TRIM8-mediated degradation of HNF1α inhibits the oncogenic effects of TRIM8 in HCC

**Fig. 7:**
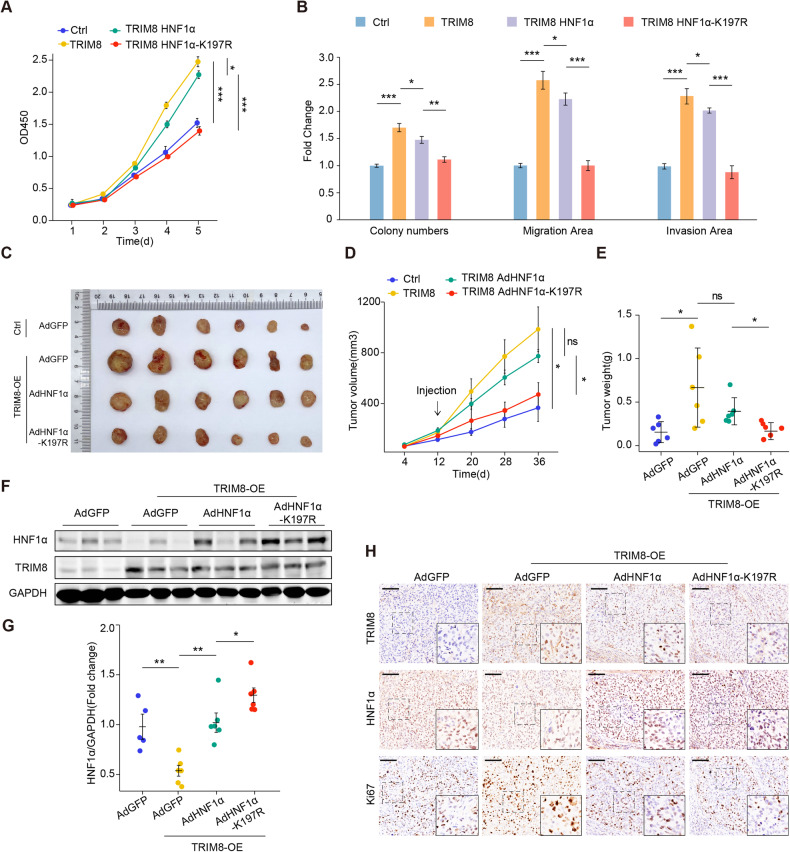
K197R mutation of HNF1α blocks TRIM8-mediated malignant properties in HCC cells. **A** CCK-8 assays of MHCC-L cells infected with control lentivirus or lenti-TRIM8 together with lentivirus expressing HNF1α or HNF1α-K197R. **B** Statistical graphs of clone formation, migration and invasion of MHCC-L cells in the indicated groups. **C** Images of xenograft tumors from mice inoculated with MHCC-L cells infected with control lentivirus or lenti-TRIM8, and were injected intratumorally with adenovirus expressing GFP, HNF1α or HNF1α-K197R. **D** Tumor volume was measured at the specified time points following subcutaneous implantation and intratumor injection. **E** Tumor weight was assessed when mice were sacrificed. **F** Representative WB analysis of HNF1α and TRIM8 protein levels in the xenograft tumors. **G** Semi-quantification analysis of HNF1α protein levels of xenograft tumors in the indicated groups. **H** The expression of HNF1α, TRIM8 and Ki67 proteins in the xenograft tumors was determined by immunohistochemistry. Scale bar = 100 μm. ns no significance, *P < 0.05, **P < 0.01, ***P < 0.001 using non-parametric Mann–Whitney test.

Proving that TRIM8 mediated degradation of HNF1α, we attempted to investigate whether blocking this degradation could suppress the effects of TRIM8 in HCC. Firstly, we infected TRIM8-overexpressed MHCC-L cells with control or HNF1α-WT or HNF1α-K197R lentivirus (Supplementary Fig. S7C, D). The promoting effects of TRIM8 on proliferation, migration and invasion were abolished by HNF1α-K197R mutant (Fig. 7A, B; Supplementary Fig. S7A, B). Then we investigate whether inhibiting the TRIM8 mediated degradation of HNF1α could reverse the oncogenic effects of TRIM8 in vivo. Nude mice were subcutaneously transplanted with HCC cells infected with TRIM8 lentivirus or control lentivirus, and received intratumoral injections of adenovirus expressing GFP (Ad-GFP), HNF1α (Ad-HNF1α) or HNF1α-K197R (Ad-HNF1α-K197R). Strikingly, overexpression of TRIM8 promoted the growth, volumes and weights of tumors, while Ad-HNF1α-K197R reversed the promoting effects of TRIM8 on the growth of tumors in mice (Fig. 7C–E). Moreover, we examined the expression of HNF1α in those xenograft tumors and found that HNF1α protein decreased in TRIM8-OE group compared with control group, while HNF1α protein level was markedly increased in AdHNF1α-K197R group compared with AdHNF1α group (Fig. 7F, G). However, the mRNA expression level showed no significant differences between AdHNF1α group and AdHNF1α-K197R group (Supplementary Fig. S7E). Immunohistochemistry (IHC) staining of Ki67 further supported that Ad-HNF1α-K197R strongly inhibited the TRIM8-induced proliferation of tumors in mice (Fig. 7H). Collectively, these results revealed that TRIM8 plays an oncogenic role in HCC by mediating the degradation of HNF1α.

## Discussion

As an E3 ligase, TRIM8 mediates the ubiquitination of diverse substrates in the different kinds of carcinoma [12]. Therefore, TRIM8 has been reported to play important but divergent roles in various types of cancer. TRIM8 promotes the degradation of oncoproteins such as MDM2 to stabilize p53 in colorectal cancer [24], catalyzes K48-linked ubiquitination and degradation of estrogen receptor-α (ERα) in breast cancer [25] and mediates degradation of EWS/FLI, an fusion oncoprotein of Ewing sarcoma [26], thus suppressing the malignant phenotypes of these cancers. Conversely, TRIM8 plays an oncogenic role through facilitating the degradation of tumor suppressor proteins or enhancing the activation of oncoproteins. It has been reported that TRIM8 cancels the negative effect of PIAS3 on STAT3 by degradation of PIAS3 and enhances Src-dependent tumorigenesis of glioblastoma [27]. TRIM8 could also mediate K63-linked ubiquitination of TAK1, hence activating TNF-α, IL-1β and NF-κB signaling, which cooperatively promote malignant phenotypes of tumor cells [28, 29]. In the liver, TRIM8 also enhances the K63-linked ubiquitination of TAK1, thus aggravating various hepatic pathophysiological process, including hepatic ischemia /reperfusion injury, hepatic steatosis and fibrosis [30–32]. However, the roles of TRIM8 in HCC remained unclear. In the present study, the augment of TRIM8 expression was observed in HCC tissues and was associated with aggressive clinical and pathological characteristics for HCC patients. Besides, ROC curve showed that TRIM8 exhibited a reliable diagnostic ability in HCC patients. Moreover, a series of in vitro and in vivo studies clarified that TRIM8 promotes various malignant behaviors of HCC cells. Analysis based on RNA-Seq and TCGA data further showed that TRIM8 participates in a series of cancer-related pathways and functions as an important regulator of EMT and cell cycle in HCC. Collectively, these data unravel the undetermined oncogenic function of TRIM8 in HCC and establish TRIM8 as a novel diagnostic and prognostic biomarker and therapeutic target for HCC patients.

As a liver-specific transcription factor, HNF1α is essential for the liver development and function maintenance [15, 16]. We have previously reported that HNF1α plays an anti-fibrotic effect through initiating the transcription of SHP-1 and repressing NF-κB and JAK/ STAT pathways [17]. We further found that HNF1α suppresses the proliferation and EMT of HCC cells by increasing p21 levels and inducing G2/M arrest, meanwhile inhibiting mTOR and TGFβ /Smads pathways [18]. It was also reported that HNF1α suppresses the occurrence and progression of HCC by inhibiting cancer-associated PPARγ, Wnt, and NF-kB signaling [33–35]. Unfortunately, although it has a powerful regulatory function in the liver, the expression of HNF1α shows a gradual decline from chronic liver diseases to HCC, thus promoting the development and progression of HCC [17–19]. However, to date, most attention has been paid to the downstream targets of HNF1α, and few studies have cracked the complicated mystery of how HNF1α is reduced in HCC, especially in the post-translational modifications. Only one study showed that HNF1α could undergo degradation via the ubiquitin-proteasome pathway in HepG2 cells [36]. In our present study, degradation and ubiquitination assay demonstrated that TRIM8 accelerates HNF1α degradation through ubiquitin-proteasome pathway. RNA-Seq and GSEA showed TRIM8 expression was associated with Wnt/β-catenin, TGF-β, PPAR signaling pathway and G2/M arrest, which is highly coincident with the biological processes involved in HNF1α in HCC. In clinical HCC specimens, the high expression of TRIM8 expression was accompanied by decreased expression of HNF1α target genes. Taken together, upregulation of TRIM8 in HCC explains, at least partially, the reason of reduced HNF1α protein expression in HCC development.

Our previous attempts to reintroduction of HNF1α using recombinant adenovirus has shown powerful treating effects of orthotopic liver tumors in mice [18], which establishes overexpression of HNF1α as a novel treatment strategy for HCC patients. The emerging mRNA delivery technologies and the development of other nucleic acid vectors may allow application of HNF1α in HCC treatment to be achieved in the near future [37, 38]. However, it is worth noting that we have demonstrated in this study that TRIM8 mediates the degradation and ubiquitination of HNF1α, and the mutation of the ubiquitination site stabilized HNF1α protein, thus enhancing the inhibitory effect of HNF1α on HCC both in vitro and in vivo. These findings raise a concern that exogenous introduction of HNF1α may be degraded partially in vivo by its negative regulator, such as TRIM8, which hampers their performance as antitumor approaches. Therefore, modifications addressing this defect, such as abrogating Lys197 ubiquitination of HNF1α, may provide a more powerful candidate for gene therapy than wild-type HNF1α. In the specific clinical application, patients with high expression of TRIM8 in HCC tissues may tend to develop drug resistance because of the degradation of wild-type HNF1α. In this case, delivery of the modified HNF1α may exert a beneficial effect on HCC patients with high expression of TRIM8. Consequently, TRIM8 could be defined as a predictive biomarker to inform treatment selection.

In conclusion, our study reveals a novel mechanism by which TRIM8 facilitates the malignant progression of HCC via K48-linked ubiquitination of HNF1α, indicating the dysregulation of TRIM8-HNF1α axis as both a potential predictor of prognosis and a promising therapeutic target for HCC. Approaches designed to manipulate this axis may improve the survival of HCC patients, especially in the recent era of targeted molecular therapy.

## Materials and methods

### Patients and clinical specimens

In this study, all patients were diagnosed with HCC by postoperative pathology, and HCC tissues were collected from the Eastern Hepatobiliary Surgery Hospital and Changzheng Hospital, Naval Medical University (Shanghai, China), and informed consent was obtained from all patients before treatment. All experiments on human samples were approved by the Ethics Committee of Naval Medical University.

### Cell construction and cell culture

TRIM8-knockout cells were produced by Cyagen company (Suzhou, China). Briefly, the ribonucleoprotein (RNP) complex consists of Cas9 protein and gRNA plasmid targeting TRIM8 were transferred into HepG2 cell via electroporation. Monoclonal cells were screened and the amplified PCR products were subjected to sequencing. Clones were classified as follows: 1) Clones with sequencing results showing no overlapping peaks and consistent with the wild type were used as control cells. 2) Clones with sequencing results showing no peaks and indel mutations not in multiples of three were considered successfully knocked out clones. Human HCC cell lines (Huh7, Hep3B, HepG2, MHCC-L, PLC and SK-Hep1), L02 cells and HEK293T cells were obtained from the Type Culture Collection of the Chinese Academy of Sciences (Shanghai, China). The cells were authenticated by STR analysis (Shanghai, China) and tested for negative mycoplasma contamination using Mycoplasma Detection Kit (Sigma-Aldrich, MP0050, Missouri, USA). The cell lines were cultured in DMEM (CM10017, Macgene, Beijing, China) supplemented with 10% fetal bovine serum (FBS, Gibco, Carlsbad, California, USA) and incubated in a humidified incubator containing 5% CO2 at 37 °C

### Animals and treatment

4-week-old adult male BALB/c nude mice were purchased from Shanghai Bikai company and maintained under specific pathogen-free conditions. All the animal experimental protocols were authorized by the Animal Care and Use Committee of Naval Medical University. The mice (*n* = 24) were randomly divided into two groups. And then MHCC-L cells (2 × 10^6^ cells resuspended in100 μl of DMEM) that infected with TRIM8 lentivirus or control virus were injected subcutaneously into the right flanks of nude mice from the two groups (*n* = 12 for each group). Twelve days after subcutaneous injection, the animals from above two groups were treated with intratumoral injection of 2 × 10^9^ pfu AdHNF1α, AdHNF1α-K197R or AdGFP, respectively, once every four days (*n* = 6 for each group). Tumor volume = width^2^ × length × 0.5.

### Real-time quantitative PCR (RT-qPCR)

Total RNA of HCC cells and tissues was extracted using Trizol reagent. Purified RNA was reverse transcribed into complementary DNA (cDNA)and then subjected to SYBR Green-based real-time PCR analysis. The expression of the target transcripts was normalized against Gapdh. The primers for these transcripts were shown in Supplementary Table 1.

### Western blotting (WB)

HCC tissues or cells lysed with 1%SDS lysis buffer containing protease and phosphatase inhibitors. Proteins were separated on 8% or 10% SDS-PAGE and transferred onto a methanol-activated NC membrane. The membrane was blocked in PBST containing 5% defatted milk for 1 h at room temperature and incubated with the corresponding primary antibodies at 4 °C overnight. Subsequently, NC membranes were incubated with the secondary antibody for 1 h. An Odyssey infrared imaging system was used to detect and photograph the protein signals. The antibodies used in current study are shown in Supplementary Table 2.

### Cell proliferation assay, migration and invasion assay

Huh7 and MHCC-L cells were plated in 96-well plates at 3× 10^3^ cells/well and cultured in DMEM containing 10% FBS. The number of metabolically active cells was determined using the Cell Counting Kit-8 (CCK-8, Dojindo, Japan) every day for approximately 1 week. In vitro migration and invasion assays were performed using Transwell chambers (BD Bioscience) without or with Matrigel. 3× 10^4^ Huh7 cells or 5× 10^4^ MHCC-L cells in serum-free DMEM were seeded in the upper chamber, and 500 μl DMEM supplemented with 10%FBS was added into the lower chamber. After incubation for 48–72 h at 37 °C, the invaded cells were fixed with 4% paraformaldehyde and stained with 0.1% crystal violet. Five fields of cells were photographed and counted to estimate cell density. Image-Pro Plus 6.0 was used to measure the stained area.

### Immunohistochemistry (IHC)

3–5-μm thick paraffin sections of HCC tissues were deparaffinized in xylene and rehydrated in graded alcohols. Endogenous peroxidase was deactivated by 0.3% H_2_O_2_ followed by EDTA antigen retrieval. Sections were blocked with 10% goat serum at 37 °C for 1 h and incubated with primary antibodies overnight at 4 °C. After washing with PBST for three times, sections were incubated with corresponding secondary antibodies. Staining was conducted using an EnVision Detection Rabbit/Mouse Kit (GK500710, GeneTech). The antibodies used in IHC are shown in Supplementary Table 2.

### RNA interference and plasmid transfection

SiRNAs were purchased from GenePharma (Shanghai, China) and plasmids were construct by YouBio (Changsha, China), siRNA and plasmid were transfected using Lipofectamine 2000 reagent (Invitrogen) according to the manufacturer’s protocols. The siRNA sequences of TRIM8 : 5’- GCAGACAGUGGAGGUCCUATT-3’.

### Virus

To overexpressing TRIM8 or HNF1α, YouBio (Changsha, China) provided lentiviral plasmid with lentiviral vectors pCDH. Lentiviral plasmid, packaging plasmid psPAX2 and envelope plasmid pMD2.G were co-transfected into HEK-293T cells in the presence of Lipofectamine 2000. After 48 h, the medium containing lentivirus was collected, lentiviral particles were concentrated and stored at −80 °C until use. Recombinant adenoviruses AdGFP and AdHNF1α were previously established in our lab. AdHNF1α(K197R) was purchased from WzBio (Shandong, China).

### Gene expression and survival analysis

The RNA-seq expression data and clinical information of LIHC tumor tissues were downloaded from the TCGA (The Cancer Genome Atlas) database. Differential gene expression analysis was conducted between cancer samples and non-tumor samples by R-package3.6.3 and ggplot2 was used to visualize the data. Gene correlation analysis was conducted using Spearman method by R-package4.2.1, and ggplot2 was used to visualize the data. HCC clinical samples were divided into two groups according to the expression of TRIM8. Then, R-package survminer was used for survival curve and data visualizing.

### RNA sequencing and the correlative analysis

Total RNA from control cells and TRIM8-KO cells was extracted using Trizol reagent (Takara). The sequencing was conducted using Illumina Novaseq6000. RNA libraries were built using standard Illumina protocols. HTSeq v0.6.0 was used to count the reads numbers mapped to each gene. DESeq2 was used to identify differentially expressed genes (DEGs) based on the criteria: |log2FC|>1 and *P* value < 0.05. Kyoto Encyclopedia of Genes and Genomes (KEGG) analysis was applied to identify the significant pathways. Gene ontology (GO) analysis was performed to reveal the biological functions of specific genes and GO categories were identified by Fisher’s exact test, and the p values were corrected by FDR. Gene Set Enrichment Analysis (GSEA) was performed by the software GSEA 4.3.2 download from http://software.broadinstitute.org/gsea/index.jsp. Gene sets used in this article was ‘h.all.v2022.1.Hs.symbols.gmt’, downloaded from the Molecular Signatures Database (MSigDB). Pathways with an absolute value of Normalized Enrichment Score (|NES|) greater than 1, a *p*-value less than 0.05, and a False Discovery Rate (FDR) less than 0.25 are generally considered to be significantly enriched. Our RNA sequencing data are accessible at NCBI Gene Expression Omnibus (http://www.ncbi.nlm.nih.gov/geo/) under the accession number GSE236348. The dataset of HNF1α-related microarray is available under the accession number GSE103128.

### Coimmunoprecipitation (Co-IP)

HEK293T cells transfected with Flag-TRIM8 or V5-HNF1α were digested using IP lysis buffer (Thermo, USA) at 4 °C for 30 min and the cell precipitate was centrifuged at 12000 g for 15 min. Then, the cell lysate was incubated with 15 μl anti-Flag affinity gel (Sigma, USA) or anti-V5 affinity (Millipore, USA) gel overnight at 4 °C. Subsequently, the affinity gel was separated from the complex by centrifuging at 3000 g for 5 min and then washed three times in wash buffer (20 mM Tris-Hcl (PH = 7.4), 100 mM NaCL, 0.5% NP40, 0.5% Triton-X100, 10% glycerol). Finally, the proteins were separated from the affinity gel-protein complex by heating with 5 × loading buffer at 100 °C for 10 min. For endogenous Co-IP, Huh7 cells were digested using IP lysis buffer and centrifuged. Then, the cell lysate was incubated with 10 μl TRIM8 antibody (sc-398878, Santa Cruz, USA) or 2 μl IgG overnight at 4 °C. The next day, 10 μl protein G agarose was added and incubated with the protein-antibody complex for 3 h at 4 °C, and the subsequent steps were identical to the exogenous Co-IP protocol. HEK293T cells transfected with Flag-TRIM8 or control plasmids were digested and supernatant was incubated with anti-Flag affinity gel. After washing, the affinity gel was sent to the National Center for Protein Science Shanghai for MS analysis. Proteins present in the control group were identified as proteins binding non-specifically.The list of the top 50 potential TRIM8-interacting proteins is shown in Supplementary Table 3.

### Ubiquitination assay

Huh7 cells transfected with TRIM8 or control plasmid were treated with 20 μM Mg132 for 8 h. Cells were incubated on ice in 500 ml RIPA lysis buffer supplemented with protease and phosphatase inhibitors for 15 min. Subsequently, the cell precipitate was centrifuged at 12000 g for 15 min. Then, the cell lysate was incubated with 5 μl HNF1α antibody or 2 μl IgG overnight at 4 °C. At the following day, 10 μl protein G were added and incubated with the cell lysate for 3 h at 4 °C. Finally, the agarose was washed for three times and boiled in 5 × loading. To detect the ubiquitination of exogenous HNF1α, Huh7 cells transfected V5- HNF1α and HA-Ub were lysed with lysis buffer (20 mM Tris-HCl (PH = 7.4), 150 mM NaCl, 1 mM EDTA, 1% SDS) supplemented with 50 mM N-ethylmaleimide (NEM) and protease phosphatase inhibitors. Cell lysates were heated at 100 °C for 10 min to dissociate protein-protein interactions and centrifuged at 12000 g for 15 min. Then, the cell lysate was incubated with 15 μl anti-Flag affinity gel or anti-V5 affinity gel (Millipore, USA) overnight at 4 °C. In the end, the affinity gel was washed for three times and boiled in 5× loading. Western blotting assay was used to assess ubiquitin.

### Dual-luciferase reporter activity assay

Cells transfected with HNF1α reporter plasmid pGL3-HNF4a-P2 and the control pRL-SV40 vector (E2261, Promega, USA) together with the plasmid overexpressing TRIM8 or the control plasmid were plated in 96-well plates. Luciferase activity was measured using the Dual-Glo Luciferase Assay System (E2920, Promega, USA) 24 or 48 h post-transfection.

### Tandem ubiquitin-binding entity (TUBE) assay

To detect the ubiquitination of HNF1α, we performed TUBE pulldown analysis using ubiquitin specific agarose TUBE (Life Sensors, USA). Huh7 cells were transfected with the plasmid overexpressing TRIM8 or the control plasmid and treated with 20 μM Mg132 for 8 h and finally harvested in RIPA lysis buffer containing 50 mM N-ethylmaleimide (NEM) and protease phosphatase inhibitors. Cell lysate was centrifuged to remove debris and quantified. For each 1 mg of protein, 10 µl of TUBE agarose was required and incubated with cell lysate overnight at 4 °C. Agarose was collected and washed 3 times in TBST, then resuspended in elution wash buffer 1 and centrifuged at 3000 g for 5 min. The beads were resuspended in elution buffer 2 and mixed for 15 min at room temperature, and the supernatant was finally separated after centrifuged (3000 g, 4 °C, 5 min). The neutralization buffer was added to neutralize the supernatant and then the samples were sent for MS analysis. The list of the top 50 identified potential substrates of TRIM8 is shown in Supplementary Table 4.

### Statistical analyses

Data analysis was performed using IBM SPSS Statistics 21 software with a two-sided test approach. Survival curves were compared using the Kaplan–Meier method with a log-rank test. The comparison of categorical variables was conducted using either a chi-square test or Fisher’s exact test. The continuous variables were firstly tested for equal variances using levene test. For data with equal variances and a normal distribution, Student’s t-test was used, and for data that did not follow a normal distribution or without equal variances, the non-parametric Mann–Whitney U test was employed. The results are presented as mean ± SEM from a minimum of three independent experiments. Statistical significance was determined at a *p*-value of less than 0.05.

### Supplementary information

Supplementary Information

Original WB

## Data Availability

The data that support the findings of this study are openly available at https://www.ncbi.nlm.nih.gov/geo/ with an accession number of GSE236348 and GSE103128.
