# Supplementary material for: Tripartite motif 8 promotes the progression of hepatocellular carcinoma via mediating ubiquitination of HNF1α
Source: Cell Death Dis. 2024 Jun 15;15(6):416. doi: 10.1038/s41419-024-06819-y (PMC11180176; doi:10.1038/s41419-024-06819-y)
Supplement: Supplementary file 1 — Supplementary Information [file 41419_2024_6819_MOESM1_ESM.docx]

**Supplementary materials**

**Supplementary Figures**


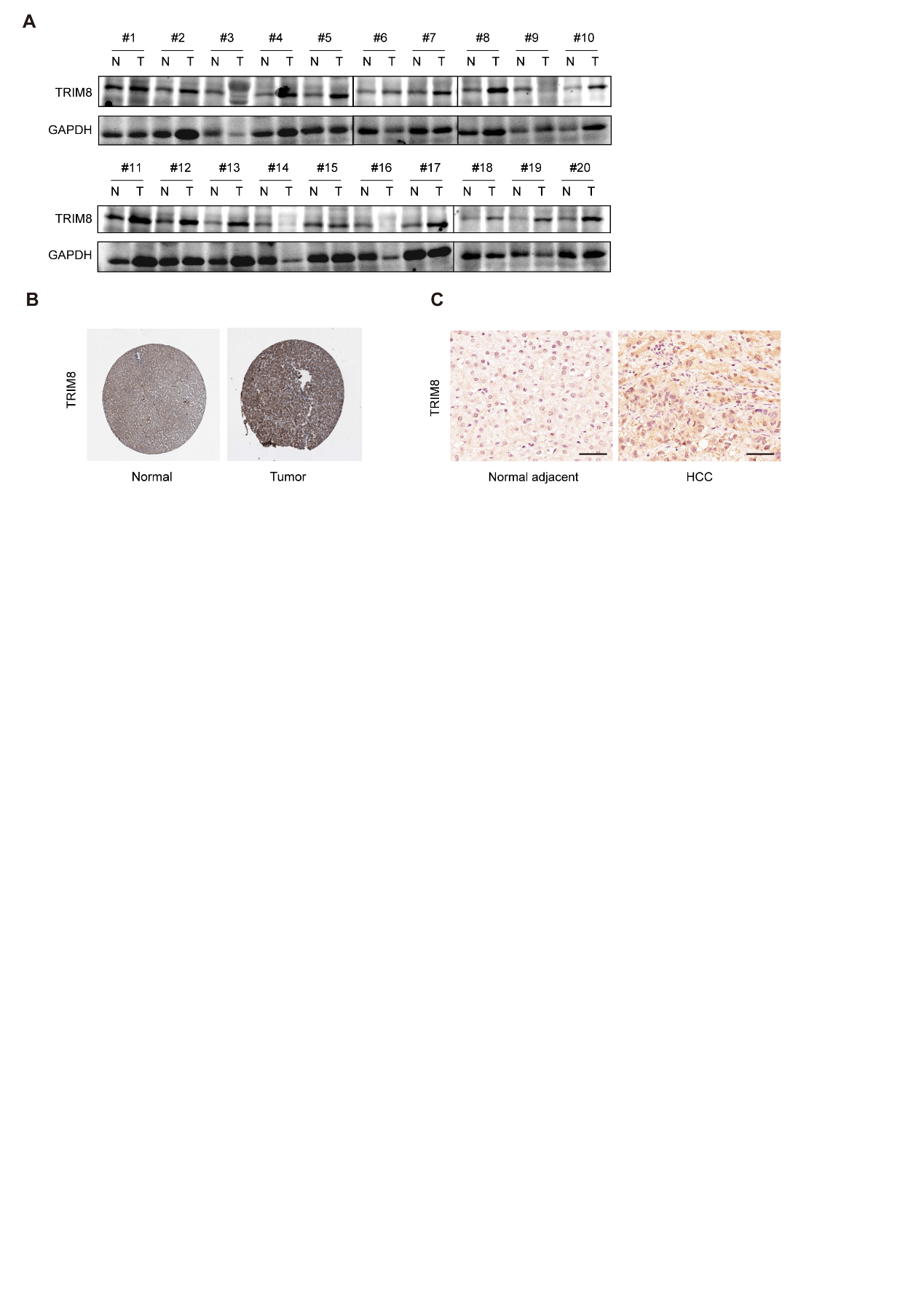


**Supplementary Figure S1. The expression of TRIM8 protein is up-regulated in human HCC.** (A) TRIM8 protein expression in 20 pairs of HCC specimens was detected by western blotting assays. (B) Representative immunohistochemistry (IHC) images of TRIM8 expression in normal (left) and HCC (right) tissues from the Human Protein Atlas. (C) Representative IHC images of TRIM8 expression in normal adjacent (left) and HCC (right) tissues from clinical samples. Scale bars = 50μm.


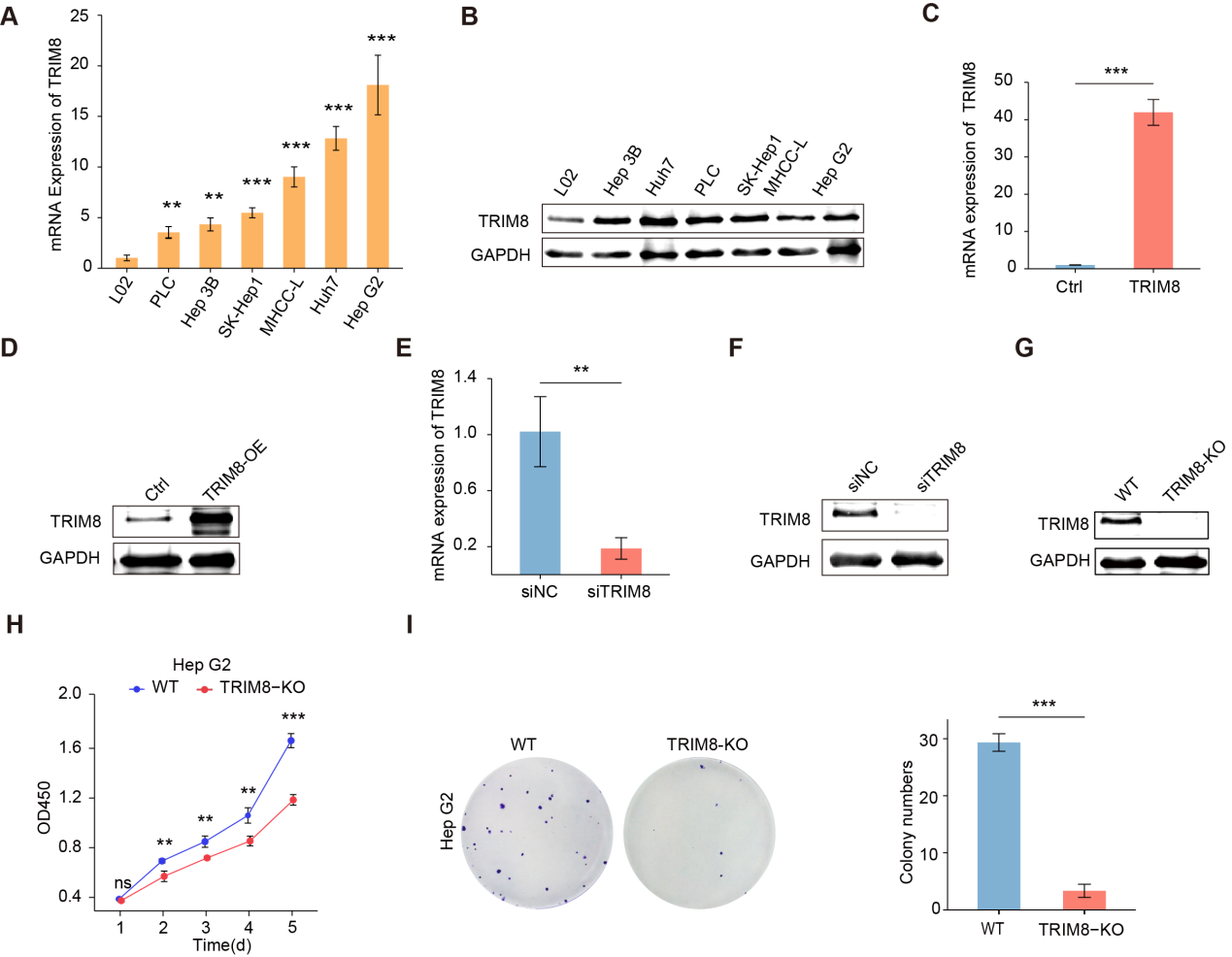


**Supplementary Figure S2. TRIM8 expression is increased in human HCC cell lines.** (A) Relative mRNA expression of TRIM8 in normal hepatocyte line L02 and six HCC lines. (B) WB analysis of TRIM8 protein levels in normal hepatocyte line L02 and six HCC lines. (C, D) Relative mRNA (C) and protein (D) expression of TRIM8 of MHCC-L cells in the indicated groups. (E, F) Relative mRNA (E) and protein (F) expression of TRIM8 in Huh7 cells in the indicated groups. (G) WB analysis of TRIM8 protein levels in wild-type (WT) and TRIM8-KO HepG2 cells. (H) Results of the CCK8 assay of the HepG2 cells in the indicated groups. (I) Representative images of colony formation (left) and its statistical results (right) of HepG2 cells in the indicated groups. Experiments were performed in triplicate and data are presented as means ± SEM. ns: no significance, ***P* < 0.01 and ****P* < 0.001 using two- tailed Student’s t- tests.


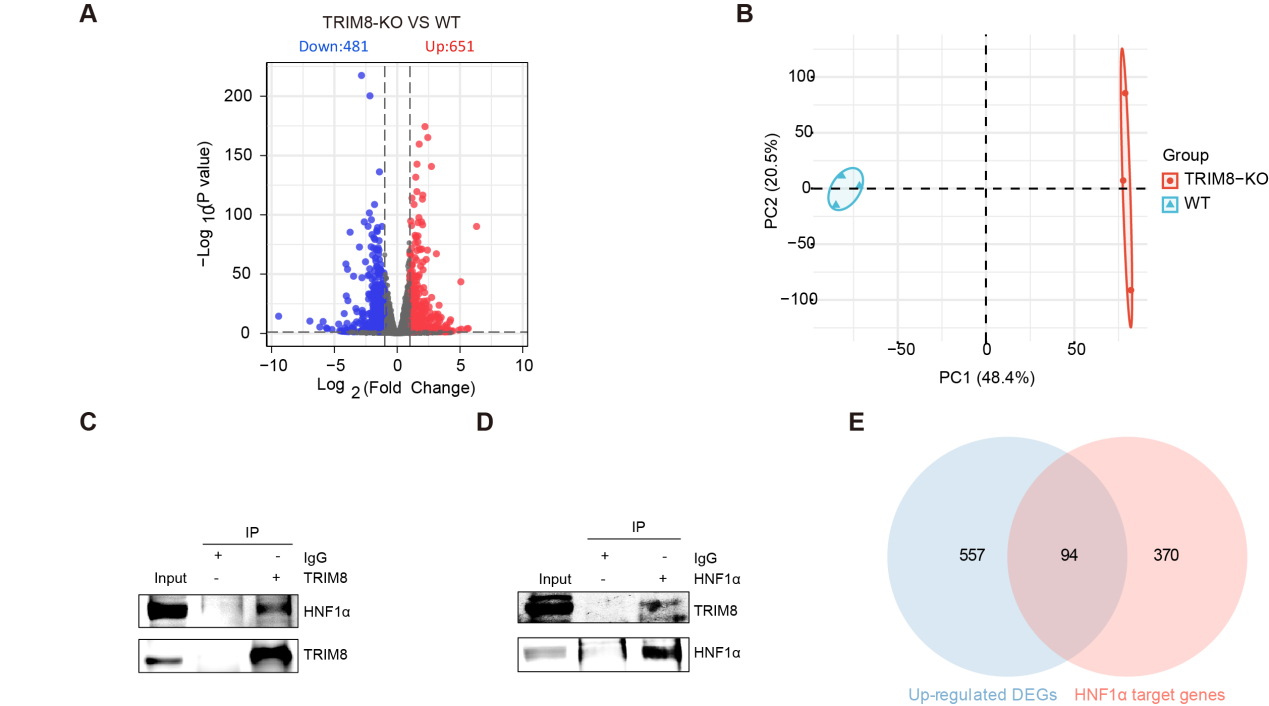


**Supplementary Figure S3. TRIM8 interacts with HNF1α.** (A) Volcano plots were used to visualize the differentially expressed genes of HepG2 cells in the indicated groups. (B) Principal Component Analysis (PCA) of transcriptomic data from HepG2 cells in the indicated groups. N = 3 samples per group. (C, D) Co-IP assays were performed to observe the interaction between endogenous HNF1α and TRIM8 in Huh7 cells using TRIM8 antibody or anti- mouse IgG to detect HNF1α(C), using HNF1α antibody or anti-rabbit IgG to detect TRIM8 (D). (E) The venn diagram of overlapping intersection between the number of HNF1α target genes and the up-regulated differentially expressed genes (DEGs) from TRIM8-KO RNA-seq.


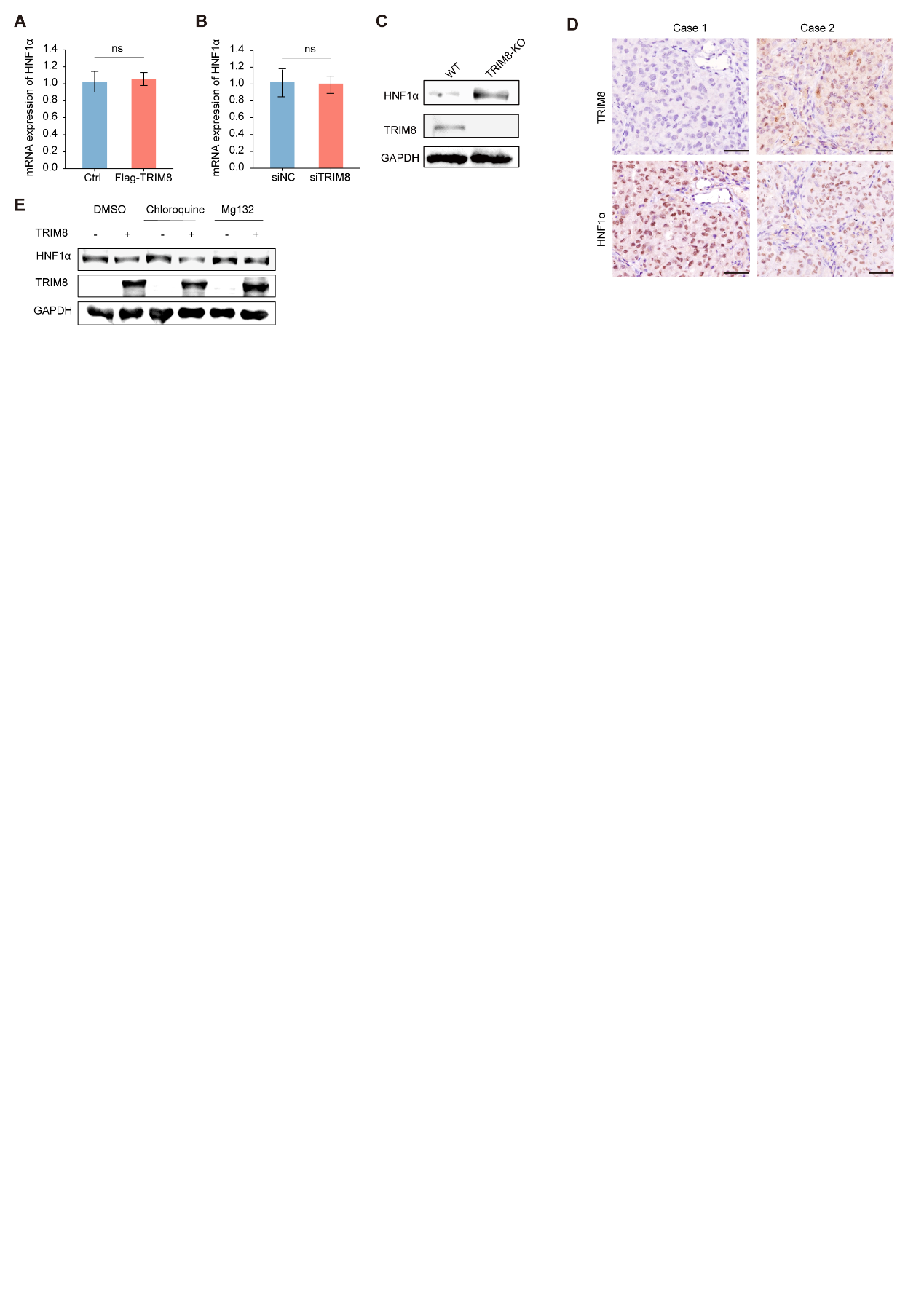


**Supplementary Figure S4. TRIM8 promotes the proteasomal degradation of HNF1α.**

(A, B) Relative mRNA expression of HNF1α in Huh7 cells transfected with control or Flag-TRIM8 plasmids (A) or transfected with control siNC or siTRIM8 (B). Experiments were performed in triplicate and data are presented as means ± SEM. ns: no significance using two- tailed Student’s t- tests. (C) WB analysis of HNF1α protein levels in WT and TRIM8-KO HepG2 cells. (D) Representative IHC images of TRIM8 and HNF1α in HCC tissues. (E) Huh7 cells transfected with TRIM8 or control plasmids were treated with MG132 (20 µM) or chloroquine (20 µM) for 4hours, and the expression of HNF1α was analyzed by WB.


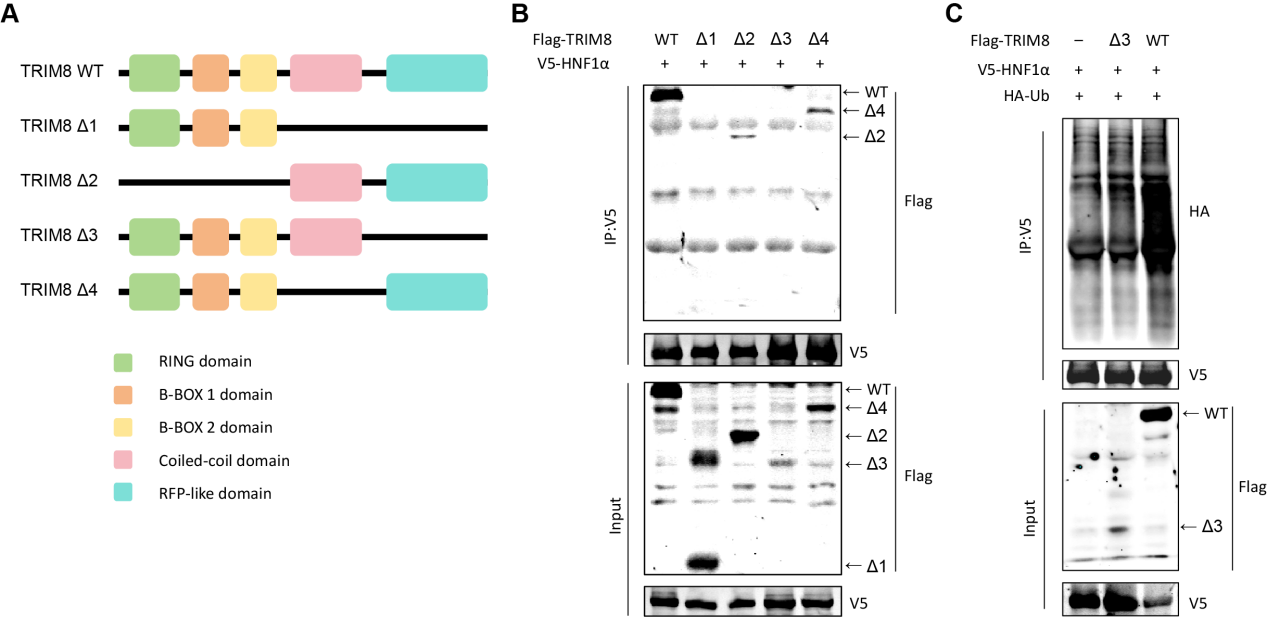


**Supplementary Figure S5. Identification of domains critical for TRIM8-mediated ubiquitination of HNF1α.** (A) Schematic of TRIM8 structural domains and mutants.(B) HEK293T cells transfected with V5- HNF1α plasmids and TRIM8 mutants for 48 h and then were lysed for immunoprecipitating, and immunoblotting with the indicated antibodies. (C) The ubiquitination levels of HNF1α were examined in Huh7 cells co-transfected with TRIM8 mutants and ubiquitin plasmids.


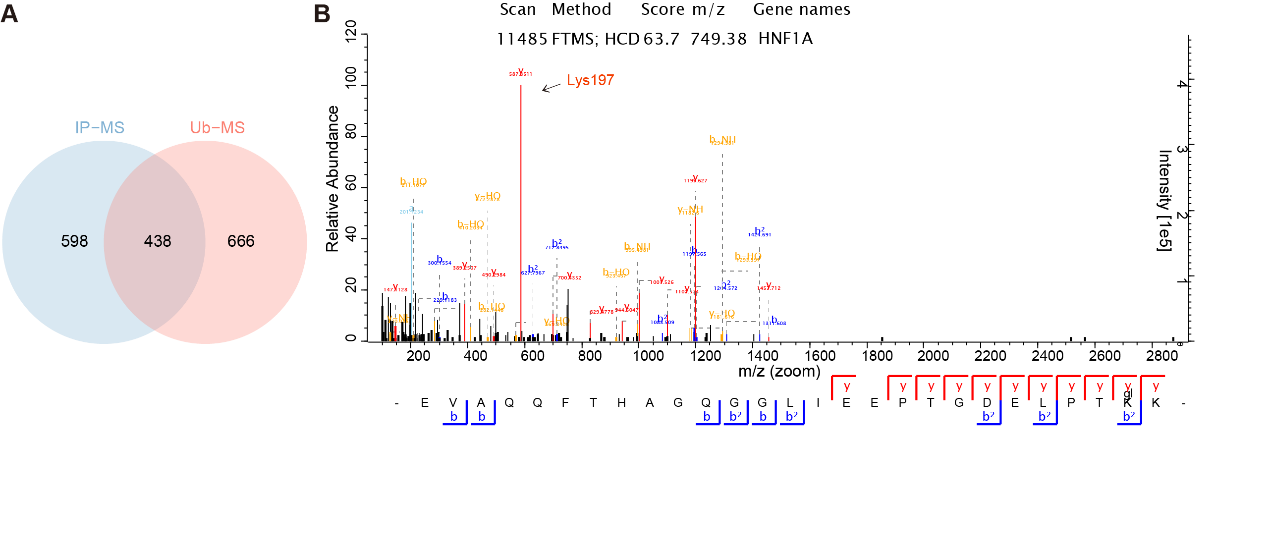


**Supplementary Figure S6. TRIM8 mediates the ubiquitination of HNF1α at Lys197.** (A) The Venn diagram of overlap number between potential TRIM8 interacting proteins (IP-MS) and ubiquitinated proteins (Ub-MS). (B) TUBE assay was used to separate ubiquitinated proteins and mass spectrometry was used to analysis the peptide, and the graph of HNF1α ion peaks was displayed.


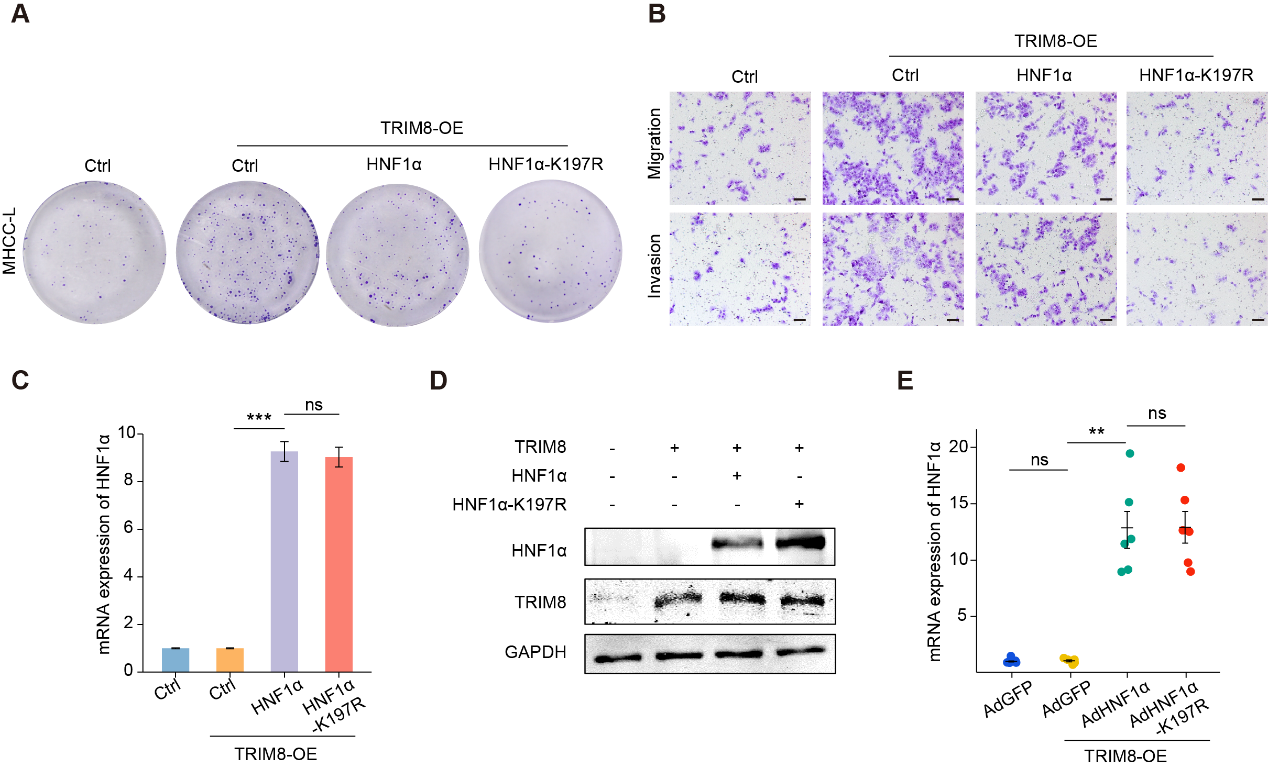


**Supplementary Figure S7. K197R mutation of HNF1α blocks TRIM8-mediated malignant phenotype in HCC cells.** (A) Images of colony formation of MHCC-L cells infected with TRIM8, HNF1α-WT, HNF1α-K197R or control lentivirus. (B) The migration (top) and invasion (bottom) of MHCC-L cells in the indicated groups. (C) HNF1α mRNA expression of MHCC-L cells in the indicated groups. (D) WB analysis of HNF1α and TRIM8 protein levels in MHCC-L cells in the indicated groups. (E) HNF1α mRNA levels of xenograft tumors in the indicated groups. Scale bars = 50 μm. ns: no significance, ***P* < 0.01, ****P* < 0.001 using non-parametric Mann- Whitney test.

**Supplementary Tables**

**Supplementary table 1. Primers used for RT-qPCR.**

| Gene(human) | Forward primer (5’-3’) | Reverse primer (5’-3’) |
| --- | --- | --- |
| HNF1A | CCATCCTCAAAGAGCTGGAG | TGTTGTGCTGCTGCAGGTA |
| TRIM8 | AAGACGGAGGATGTCAGCTTC | GTGGCCGATCTTAGTGGGG |
| GAPDH | TTGATTTTGGAGGGATCTCG | GAGTCAACGGATTTGGTCGT |
| MMP9 | TCTATGGTCCTCGCCCTGAA | CATCGTCCACCGGACTCAAA |
| MMP13 | TTCGGCCACTCCTTAGGTCT | GGTCTTCATCTCCTGGACCATA |
| SNAI1 | CGAGTGGTTCTTCTGCGCTA | CTGCTGGAAGGTAAACTCTGGA |
| Vimentin | GCTAACCAACGACAAAGCCC | GACGCATTGTCAACATCCTGT |
| N-Cadherin | AATGGATGAAAGACCCATCC | GTCATATGGTGGAGCTGTGG |
| E-Cadherin | GGCTGGACCGAGAGAGTTTC | CGACGTTAGCCTCGTTCTCA |
| ALDOB | AGGAGGACTCTTCTCTCCCAA | GATTCATCTGCAGCCAGGAT |
| G6P | GGCTCCATGACTGTGGGATC | TTCAGCTGCACAGCCCAGAA |
| GYS2 | CCAGTGGGAAGTCGAAGAAC | TTCTCTCCCCATTCATCTGC |
| TTR | TCAGAAAGGCTGCTGATGAC | AGTCGTTGGCTGTGAATACC |
| APOC3 | GGGTACTCCTTGTTGTTGC | AAATCCCAGAACTCAGAGAAC |

**Supplementary table 2. Antibodies used for WB, IP and IHC**

| Antibody | Cat No. | Manufacturer | Application |
| --- | --- | --- | --- |
| TRIM8 | 27463-1-AP | Proteintech | WB, IHC |
| TRIM8 | sc-398878 | Santa cruz | IP |
| HNF1α | 96777 | Abcam | WB, IP, IHC |
| HNF1α | HPA035231 | Sigma | WB |
| HNF1α | sc-393925 | Santa cruz | WB |
| GAPDH | 60004-1-Ig | Proteintech | WB |
| GAPDH | BSAP0063 | Bioworld | WB |
| Flag | F7425 | Sigma | WB |
| HA | H6908 | Sigma | WB |
| V5 | ab27671 | Abcam | WB |
| Ub | 20326 | Cell Signaling Technology | WB |
| Ki67 | ab16667 | ABclonal | IHC |

**Supplementary table 3. The top 50 potential TRIM8-interacting proteins in HEK293T cells derived from IP-MS.**

|  | Gene names | Number of unique peptides | Intensity | DOI of reported literature |
| --- | --- | --- | --- | --- |
| 1 | KIF11 | 59 | 13054000000 | 10.1016/j.canlet.2019.12.042 |
| 2 | TUBB | 4 | 5524400000 |  |
| 3 | TRIM21 | 22 | 5083800000 | 10.1038/s41388-023-02879-0 |
| 4 | PRMT5 | 26 | 3576700000 |  |
| 5 | TUBA1C | 1 | 2630000000 |  |
| 6 | FASN | 83 | 2378400000 |  |
| 7 | TRIM8 | 17 | 2068000000 |  |
| 8 | ENO1 | 22 | 1588100000 |  |
| 9 | RAN | 11 | 1553500000 |  |
| 10 | EIF4A1 | 20 | 1541700000 |  |
| 11 | NPM1 | 12 | 1525400000 |  |
| 12 | CCT6A | 22 | 1454900000 |  |
| 13 | HNRNPK | 22 | 1375600000 |  |
| 14 | RPL13 | 13 | 1343000000 |  |
| 15 | WDR77 | 6 | 1210840000 |  |
| 16 | HNRNPM | 40 | 1130100000 |  |
| 17 | PRMT1 | 13 | 1118740000 |  |
| 18 | RPL24 | 8 | 1036320000 |  |
| 19 | ACTG1 | 5 | 1036200000 |  |
| 20 | HSP90AB1 | 11 | 1033000000 | 10.1016/j.bbamcr.2011.05.013 |
| 21 | HSPA1B | 10 | 976100000 |  |
| 22 | DDX21 | 25 | 949120000 |  |
| 23 | DHX9 | 36 | 921800000 |  |
| 24 | PRPF8 | 55 | 876000000 |  |
| 25 | EIF4B | 33 | 853100000 |  |
| 26 | RPS24 | 4 | 841780000 |  |
| 27 | RPS10 | 11 | 840520000 |  |
| 28 | RPL15 | 15 | 808900000 |  |
| 29 | RPS7 | 7 | 803220000 |  |
| 30 | TCP1 | 19 | 778800000 |  |
| 31 | CCT5 | 23 | 774100000 |  |
| 32 | XRCC5 | 18 | 771520000 |  |
| 33 | **HNF1A** | 11 | 752320000 |  |
| 34 | EIF4A3 | 14 | 744280000 |  |
| 35 | CLTC | 42 | 732740000 |  |
| 36 | PPIA | 7 | 712820000 |  |
| 37 | EEF1G | 19 | 697800000 |  |
| 38 | RPS15 | 6 | 692800000 |  |
| 39 | SNRPD1 | 4 | 669470000 |  |
| 40 | ROCK1 | 35 | 647280000 |  |
| 41 | CFL1 | 9 | 644940000 |  |
| 42 | HNRNPA1 | 11 | 629940000 |  |
| 43 | DDX5 | 20 | 623500000 |  |
| 44 | SFPQ | 17 | 588850000 |  |
| 45 | SNRNP200 | 53 | 577420000 |  |
| 46 | RPS27 | 3 | 572420000 |  |
| 47 | STK38L | 13 | 565580000 |  |
| 48 | PHGDH | 12 | 564340000 |  |
| 49 | SLC25A6 | 4 | 557600000 |  |
| 50 | CCT3 | 22 | 549000000 |  |

**Supplementary table 4. The top 50 potential substrates of TRIM8 have been identified using TUBE and MS techniques.**

|  | Gene names | Number of unique peptides | Intensity |  |
| --- | --- | --- | --- | --- |
| 1 | UBQLN1 | 1 | 5573180000 |  |
| 2 | SUMO3 | 2 | 4248200000 |  |
| 3 | BAG6 | 37 | 2708130000 |  |
| 4 | PSMA1 | 17 | 1424500000 |  |
| 5 | PSMC2 | 24 | 1319360000 |  |
| 6 | PSMC1 | 22 | 1172540000 |  |
| 7 | PSMA7 | 12 | 1152790000 |  |
| 8 | UBQLN4 | 5 | 939796000 |  |
| 9 | HSPA1B | 9 | 935110000 |  |
| 10 | PSMB1 | 8 | 931380000 |  |
| 11 | PSMC6 | 14 | 910740000 |  |
| 12 | PSMC3 | 24 | 898420000 |  |
| 13 | KRT18 | 18 | 886680000 |  |
| 14 | APOB | 63 | 875870000 |  |
| 15 | TUBA1C | 12 | 859280000 |  |
| 16 | HSP90AB1 | 14 | 846150000 | 10.1016/j.bbamcr.2011.05.013 |
| 17 | HNRNPM | 29 | 820680000 |  |
| 18 | TRIM8 | 13 | 795630000 |  |
| 19 | HSPA5 | 28 | 795480000 |  |
| 20 | PSMA4 | 8 | 762090000 |  |
| 21 | PSMA2 | 11 | 707451000 |  |
| 22 | PSMA3 | 11 | 686200000 |  |
| 23 | PSMC5 | 25 | 645110000 |  |
| 24 | PSMA5 | 9 | 644090000 |  |
| 25 | EEF1A1 | 9 | 643560000 |  |
| 26 | FASN | 42 | 618888000 |  |
| 27 | PSMC4 | 26 | 609450000 |  |
| 28 | TUBB4B | 2 | 607710000 |  |
| 29 | PSMB3 | 7 | 582150000 |  |
| 30 | RAN | 4 | 551510000 |  |
| 31 | HSPA8 | 15 | 498060000 |  |
| 32 | HECTD1 | 45 | 475880000 |  |
| 33 | ENO1 | 11 | 471880000 |  |
| 34 | PSMB5 | 10 | 434220000 |  |
| 35 | EIF4A1 | 16 | 432427000 |  |
| 36 | SQSTM1 | 9 | 410620000 |  |
| 37 | VCP | 25 | 377411000 |  |
| 38 | PSMB4 | 6 | 367910000 |  |
| 39 | PKM | 18 | 340530000 |  |
| 40 | PSMA6 | 7 | 336380000 |  |
| 41 | PSMB7 | 6 | 307970000 |  |
| 42 | EEF1G | 11 | 290570000 |  |
| 43 | SLC3A2 | 15 | 276835000 |  |
| 44 | HNF1A | 11 | 273771000 |  |
| 45 | ACTG1 | 6 | 263400000 |  |
| 46 | HIF1A | 15 | 263334000 |  |
| 47 | PSMD11 | 11 | 262965000 |  |
| 48 | GET4 | 8 | 257310000 |  |
| 49 | RPS3A | 11 | 252580000 |  |
| 50 | IDH1 | 14 | 250980000 |  |
