# Supplementary material for: Tripartite motif 8 promotes the progression of hepatocellular carcinoma via mediating ubiquitination of HNF1α
Source: Cell Death Dis. 2024 Jun 15;15(6):416. doi: 10.1038/s41419-024-06819-y (PMC11180176; doi:10.1038/s41419-024-06819-y)

Fig.4B

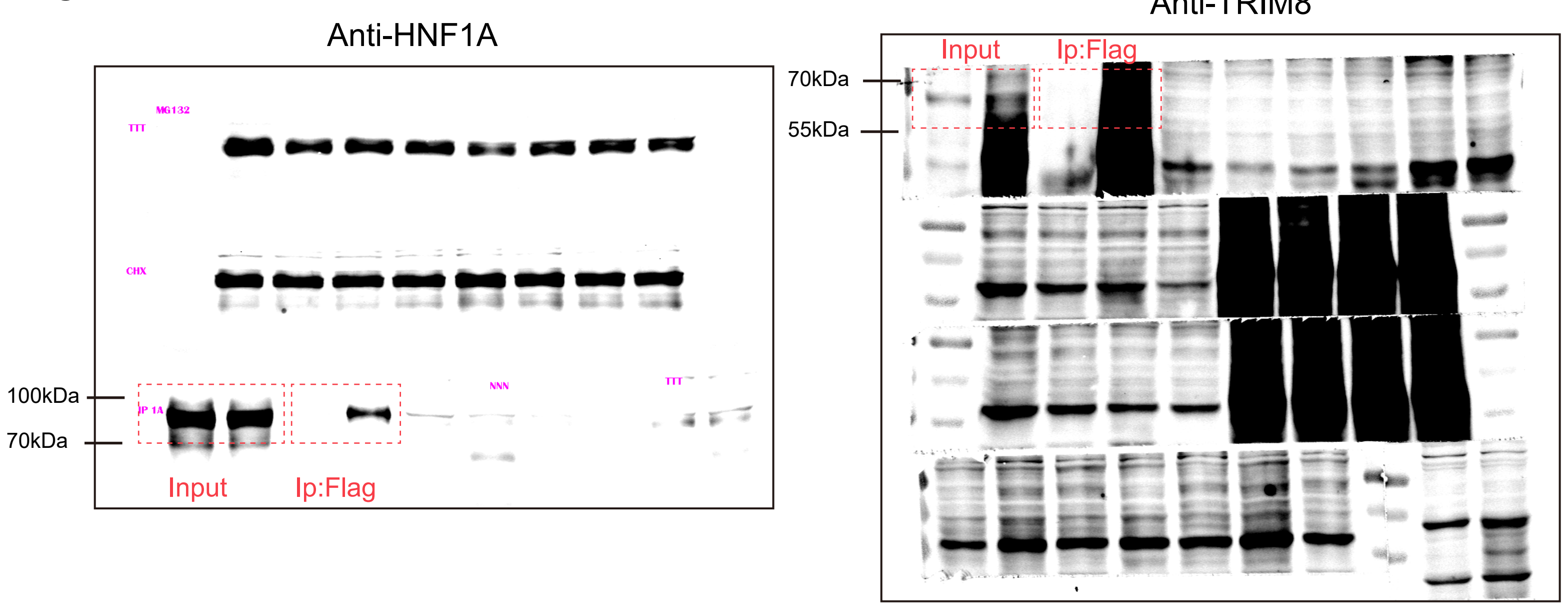

Fig.4C

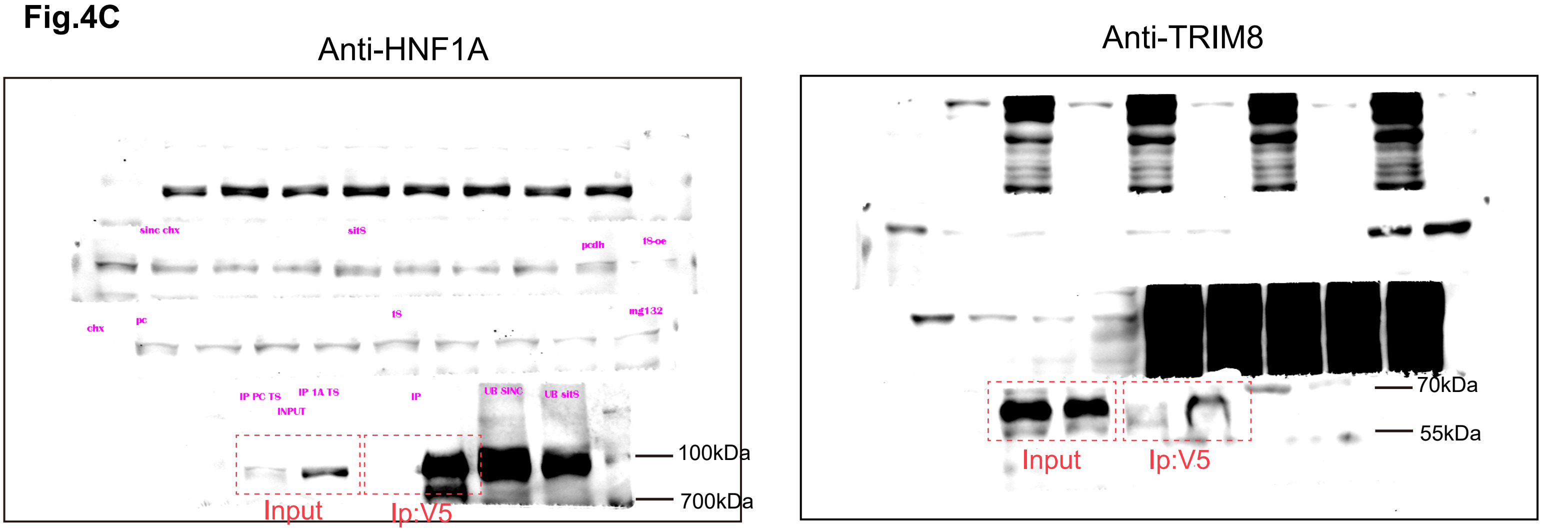

Fig.5A

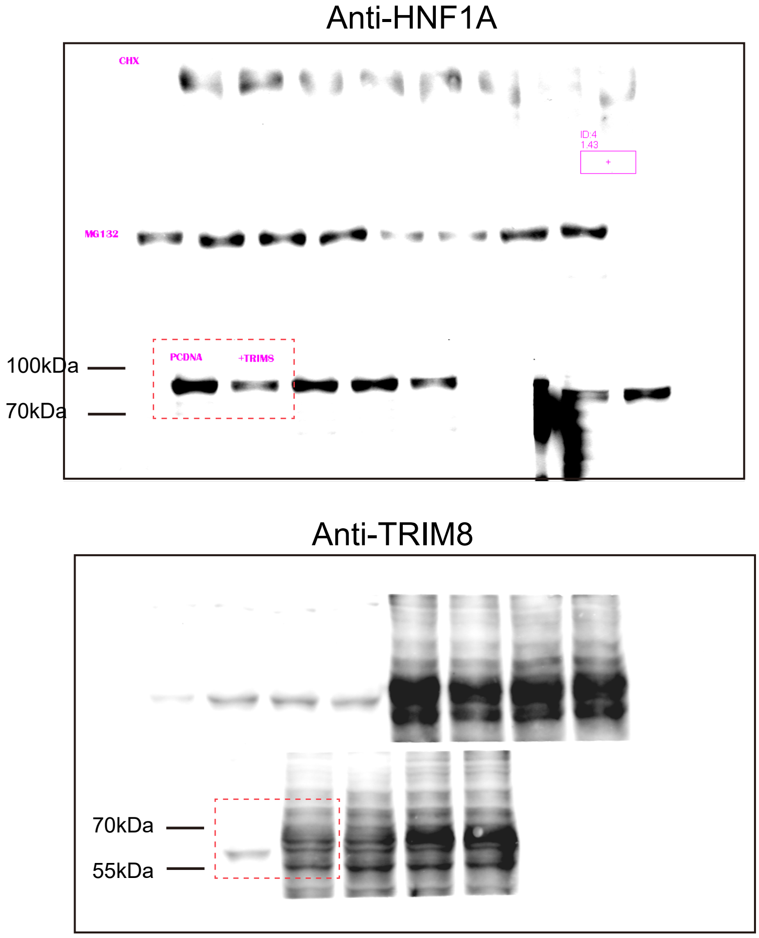

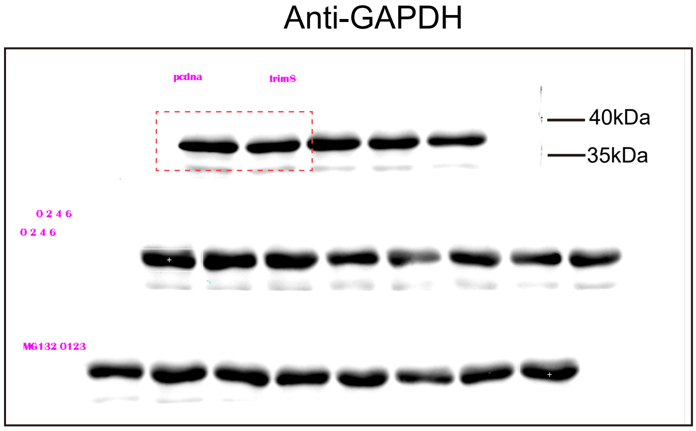

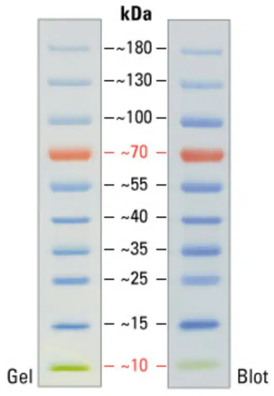

4-20% Tris-glycine gel (SDS-PAGE)

Fig.5B

Anti-HNF1A

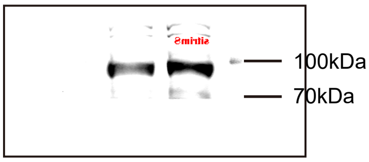

Anti-TRIM8

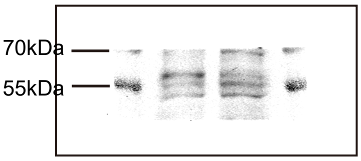

Anti-GAPDH

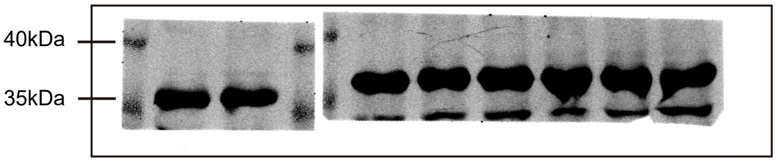

Fig.5C

Anti-HNF1A

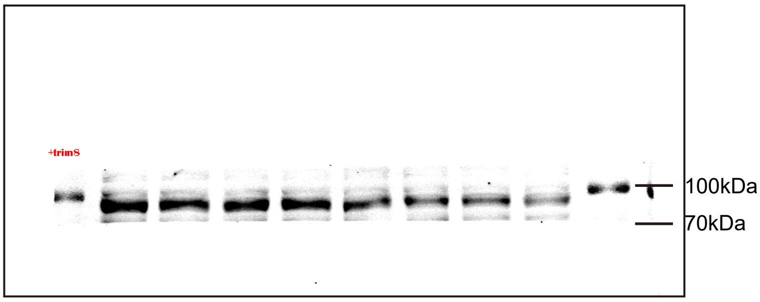

Anti-TRIM8

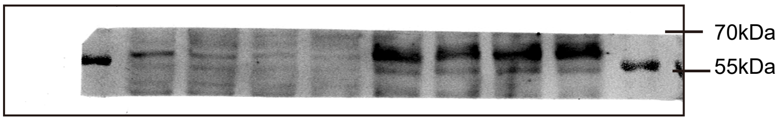

Anti-GAPDH

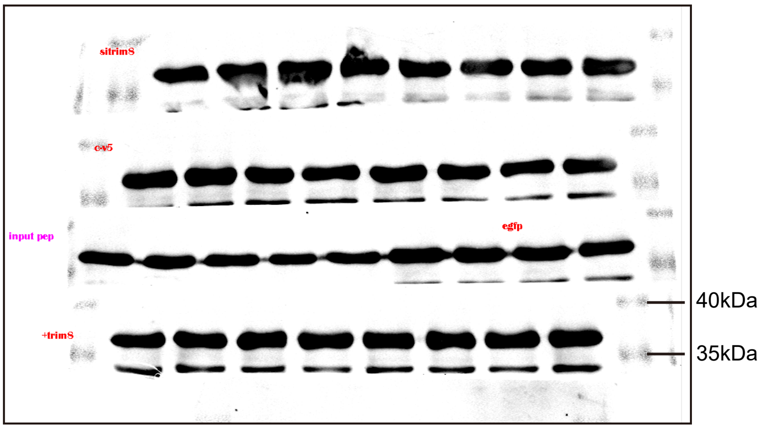

Fig.5E

Anti-TRIM8

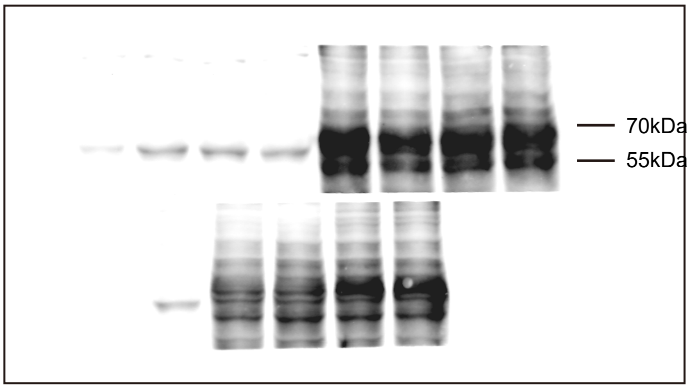

Anti-HNF1A

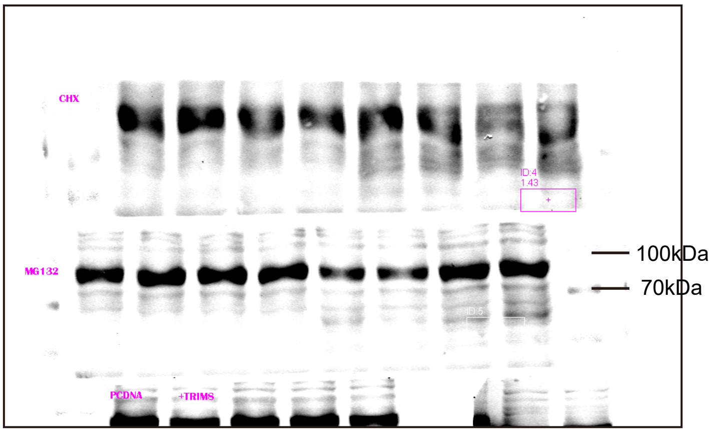

Anti-GAPDH

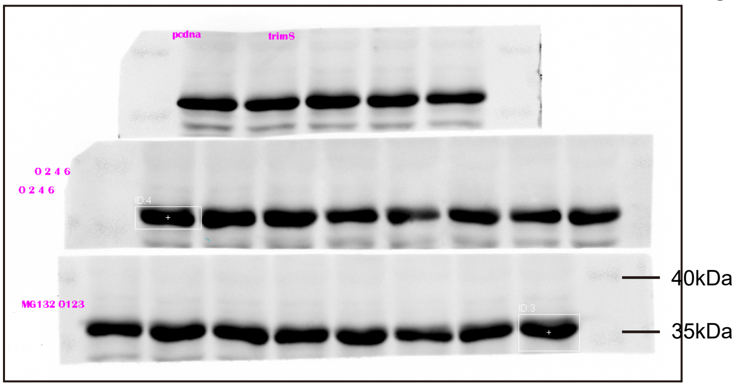

Fig.5F

Anti-GAPDH

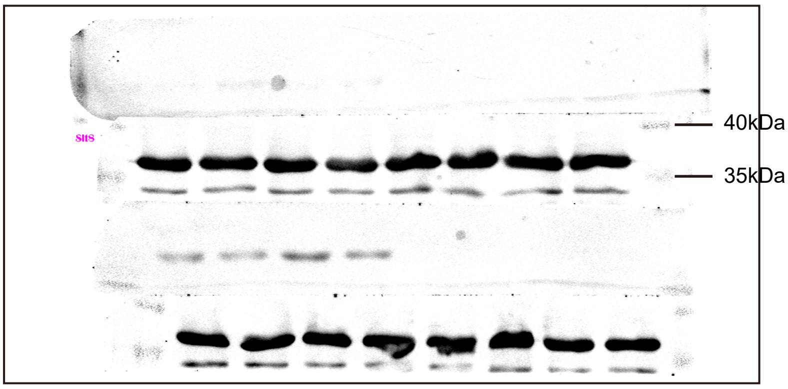

Anti-HNF1A

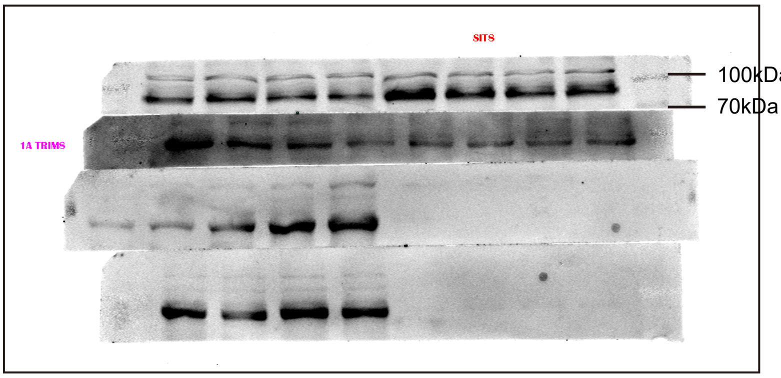

Anti-TRIM8

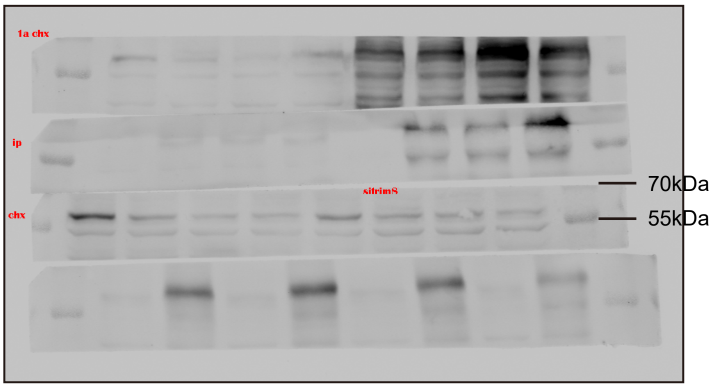

Fig.5H

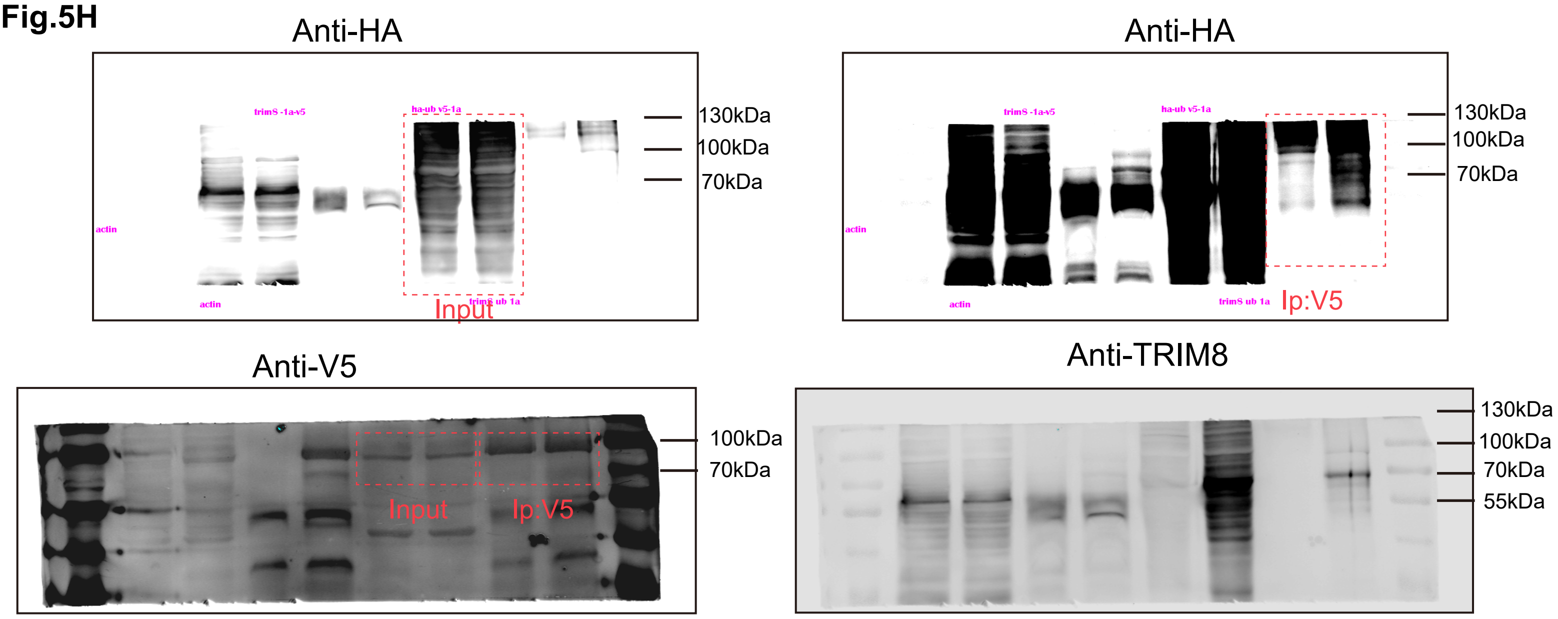

Fig.5I

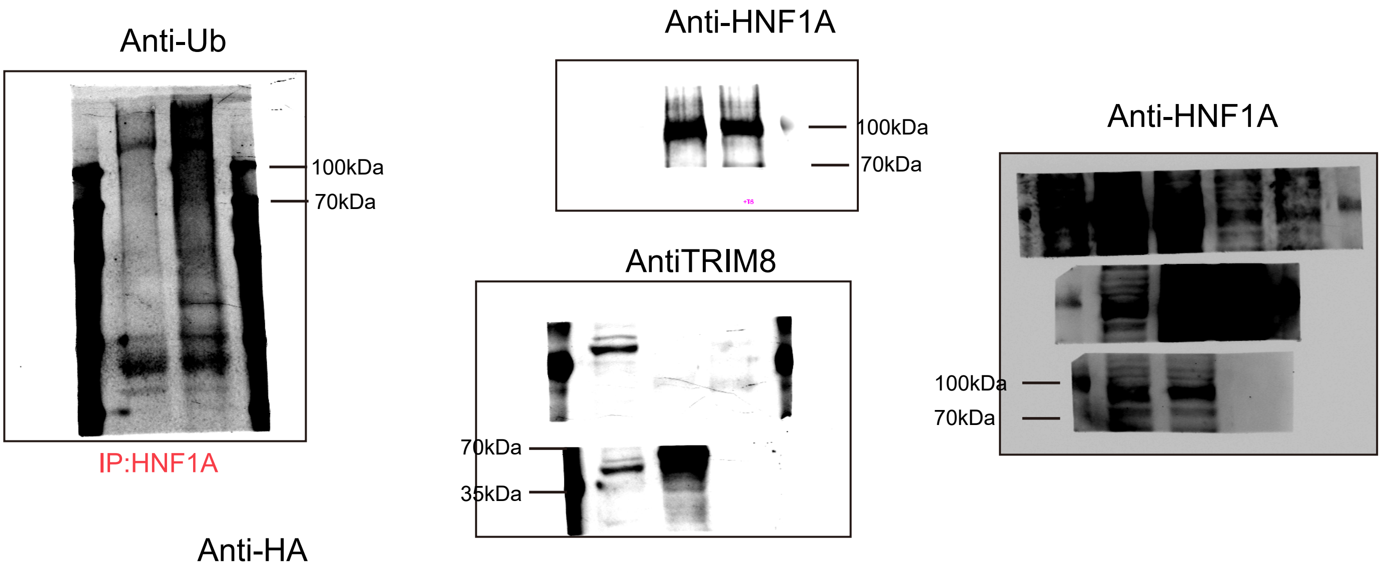

Fig.5I

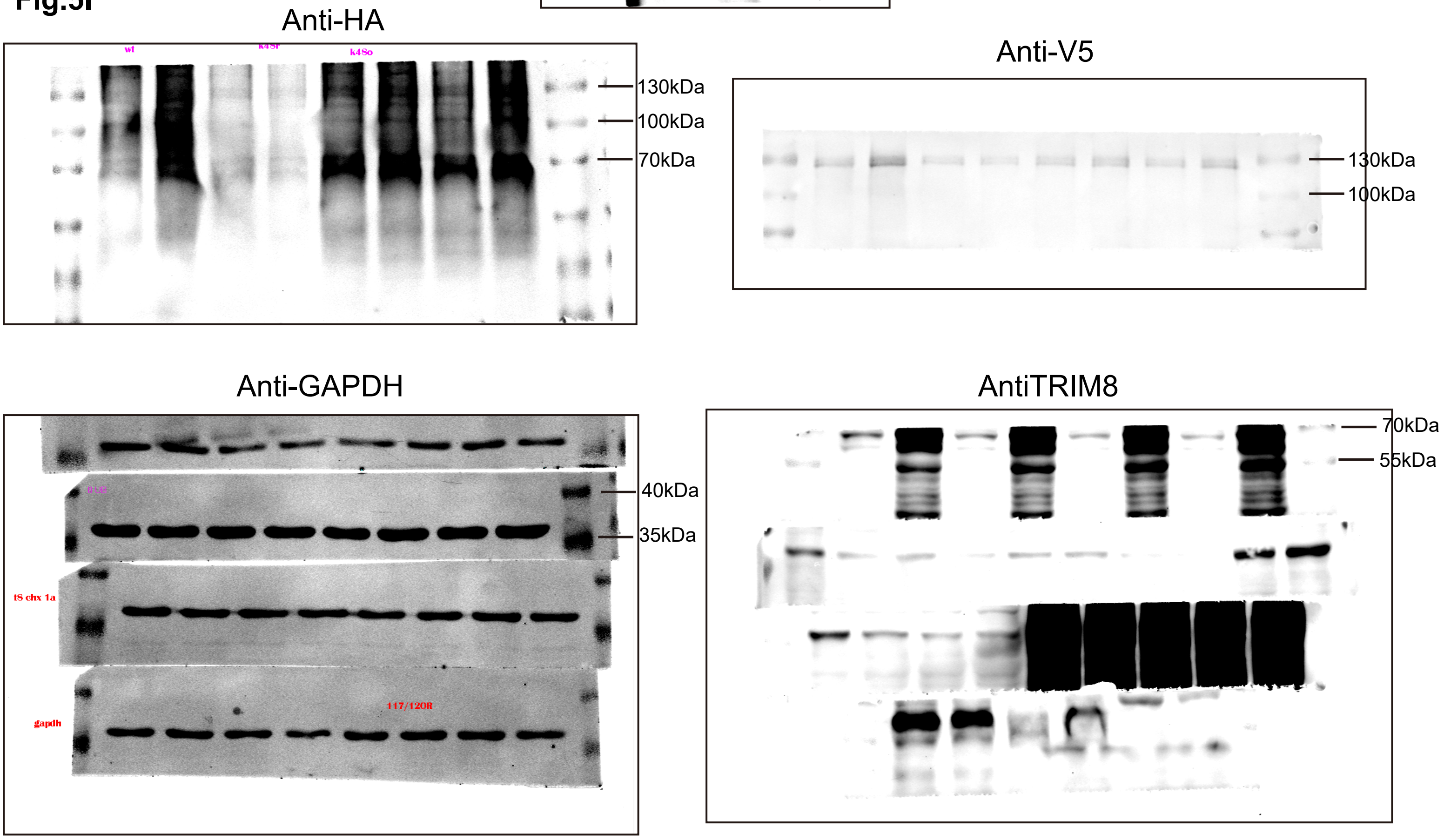

Fig.6B

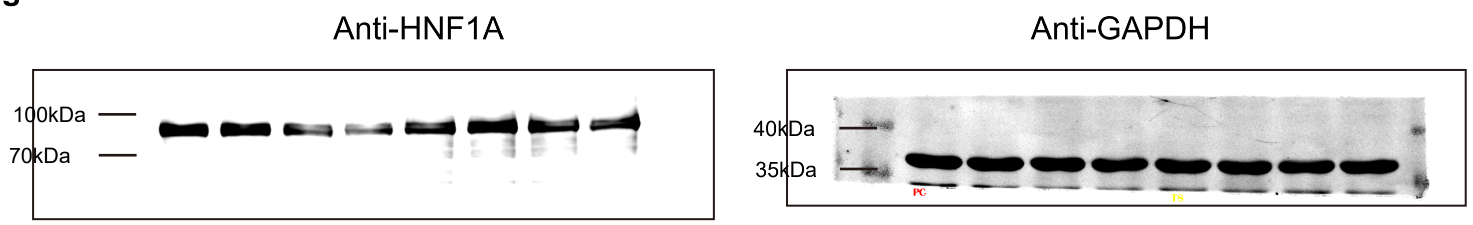

Fig.6D

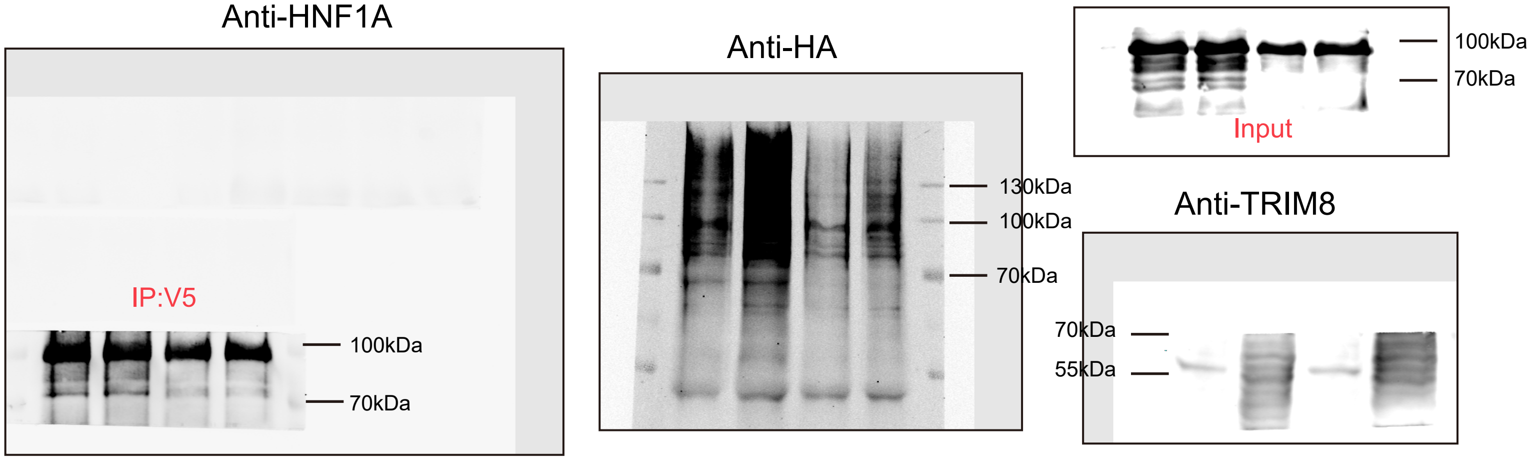

Fig.6E

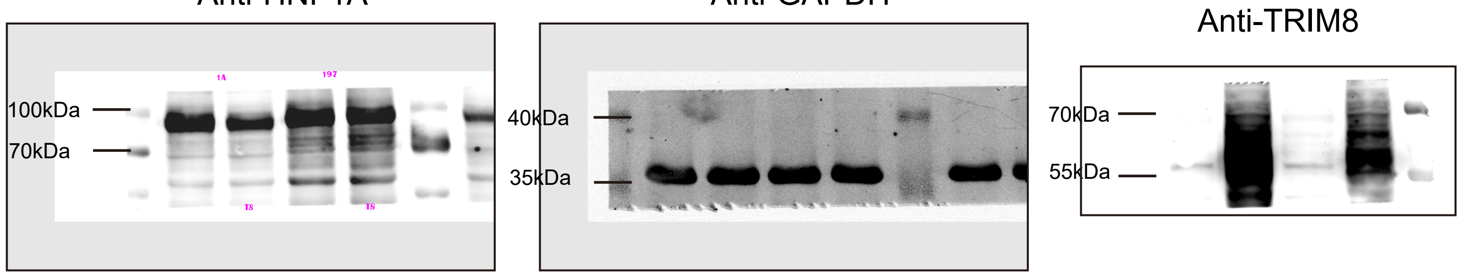

Fig.7E

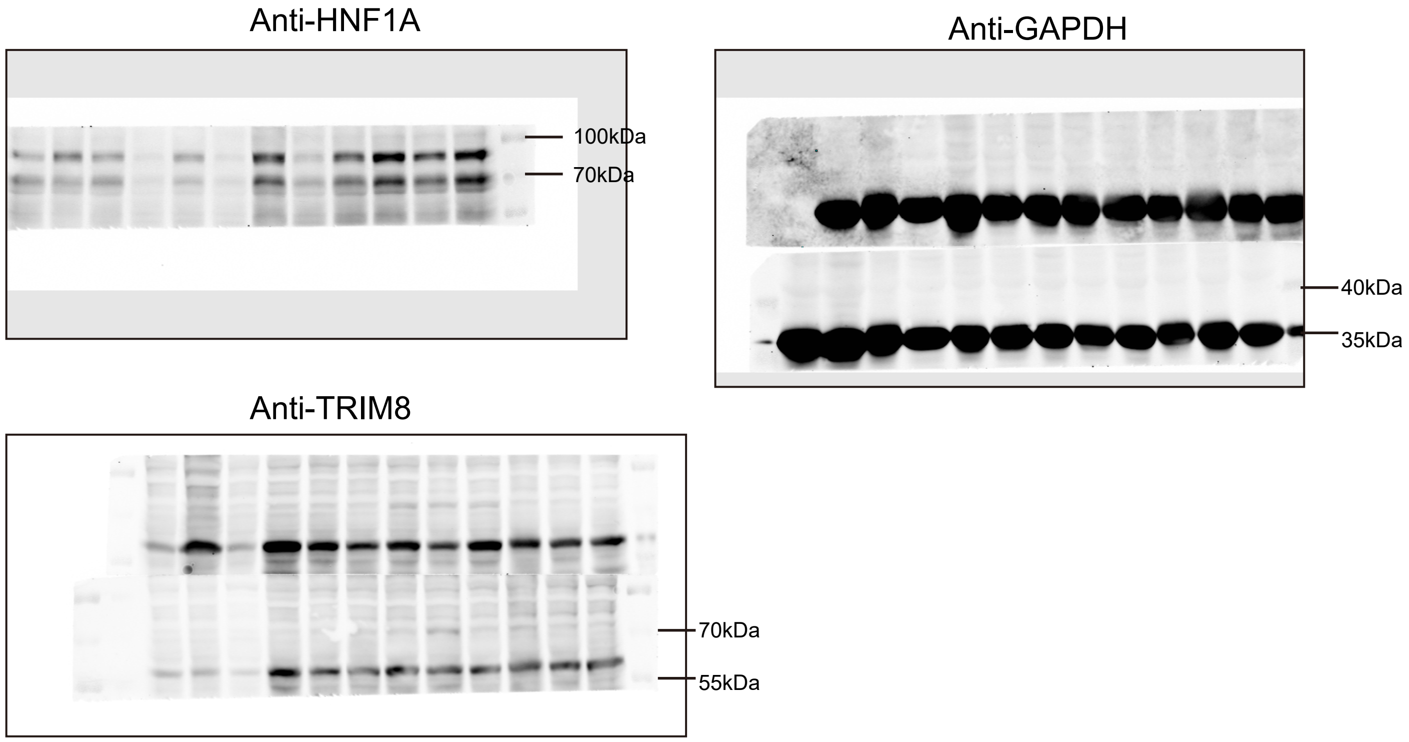

Fig.S1A

Anti-GAPDH

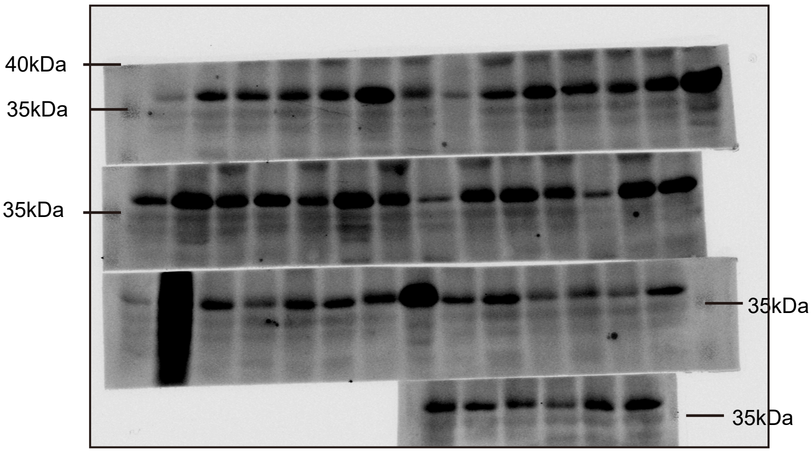

Anti-TRIM8

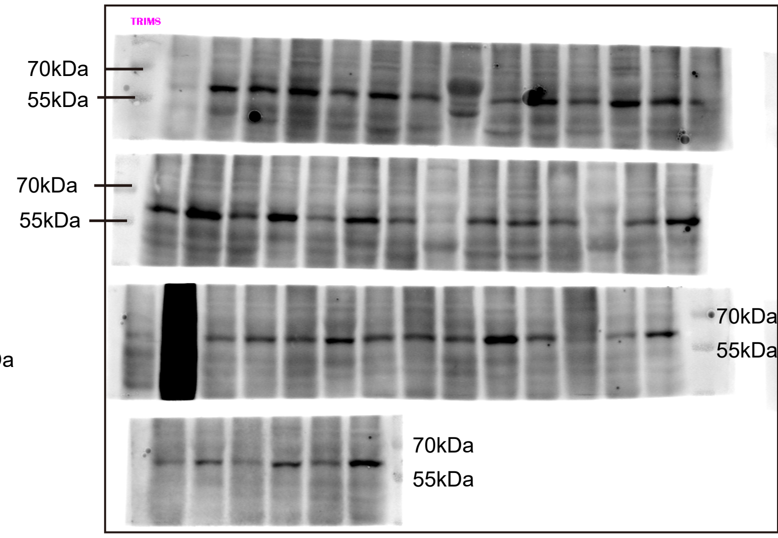

Fig.S2B

Anti-GAPDH

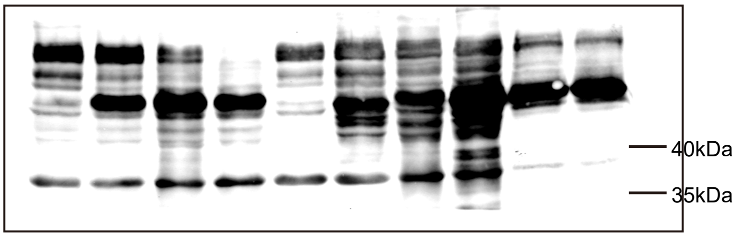

Anti-TRIM8

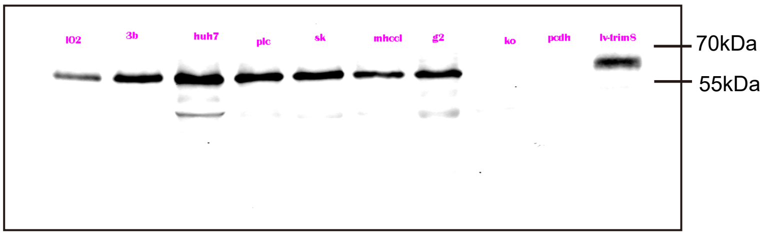

Fig.S2D,F,G

Anti-GAPDH

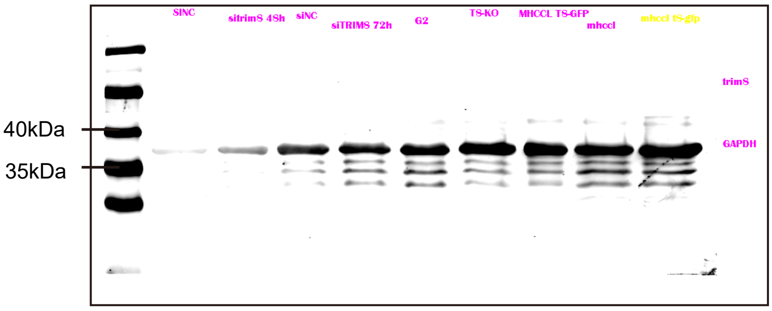

Anti-TRIM8

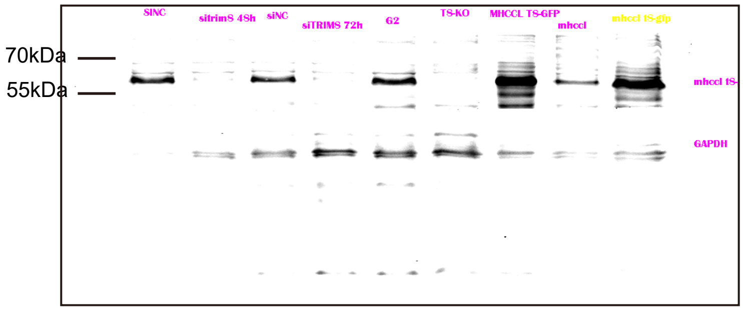

Fig.S3A

Anti-TRIM8

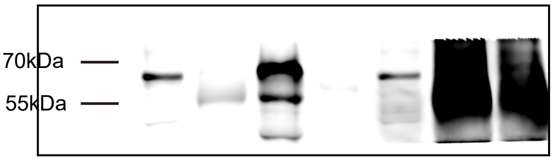

Anti-HNF1A

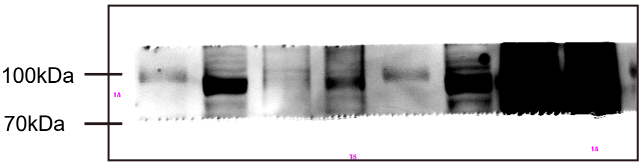

Fig.S3B

Anti-HNF1A

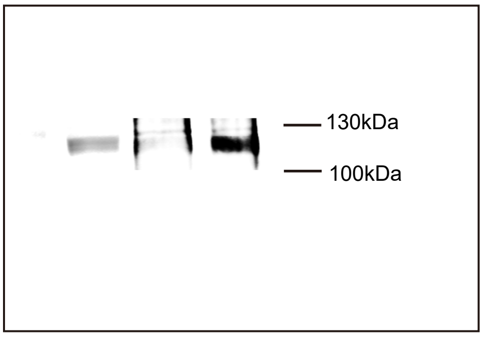

Anti-TRIM8

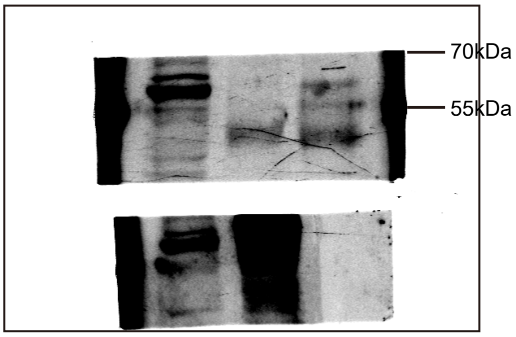

Fig.S4E

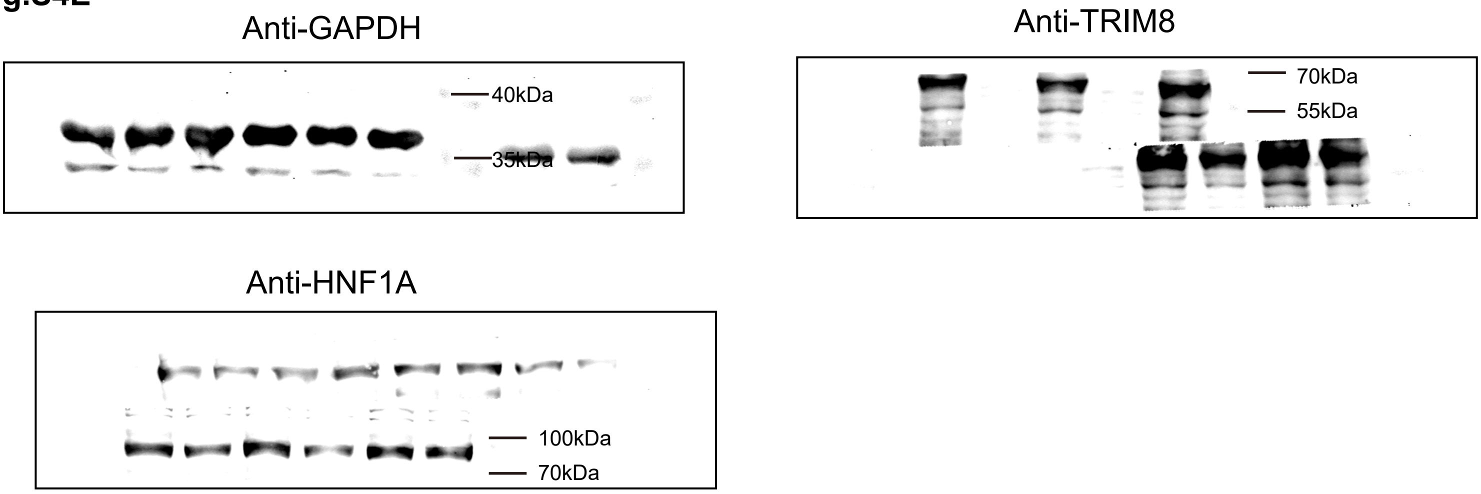

Fig.S5B

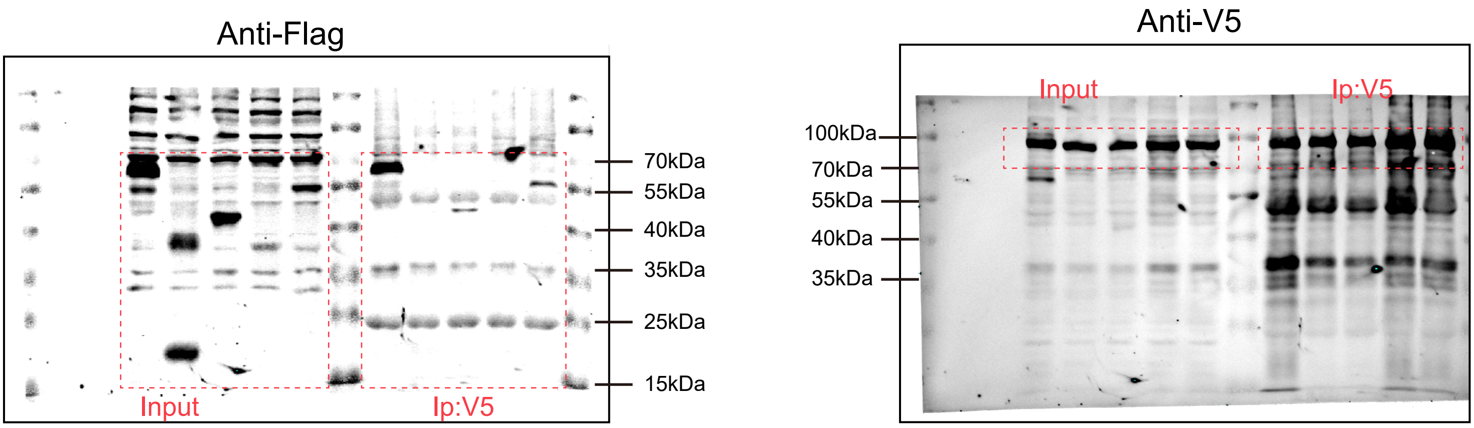

Fig.S5C

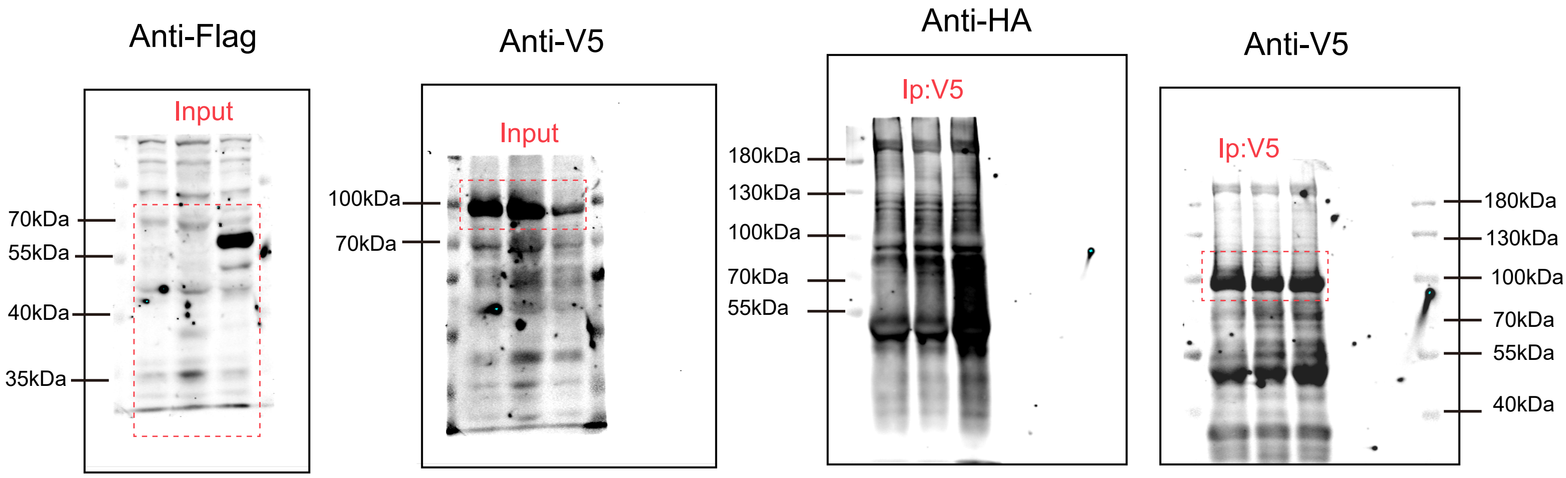

Fig.S7D

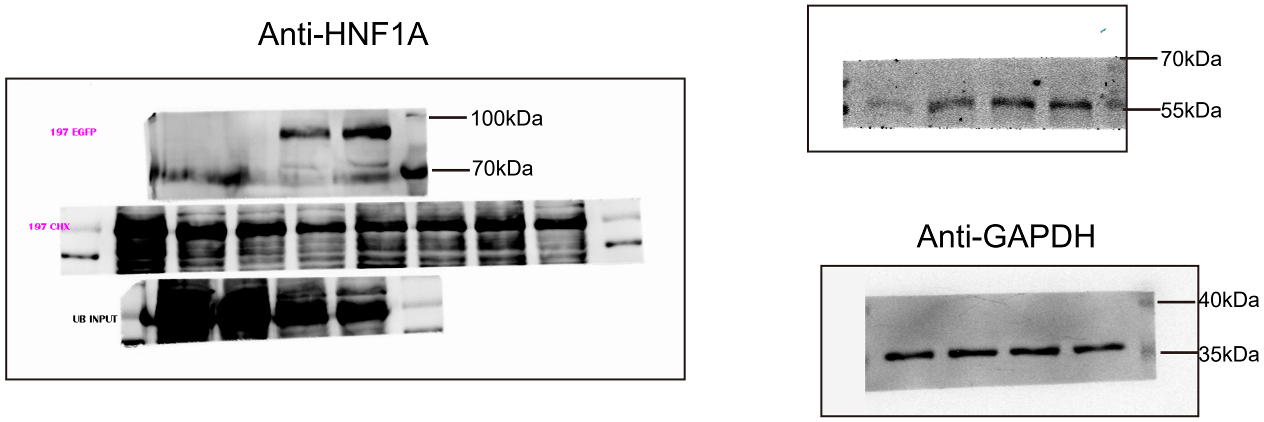

Supplement: Supplementary file 2 — Original WB [file 41419_2024_6819_MOESM2_ESM.pdf]
